# Genetic and pharmacological inhibition of the nuclear receptor RORα regulates T_H_17 driven inflammatory disorders

**DOI:** 10.1038/s41467-020-20385-9

**Published:** 2021-01-04

**Authors:** Ran Wang, Sean Campbell, Mohammed Amir, Sarah A. Mosure, Molly A. Bassette, Amber Eliason, Mark S. Sundrud, Theodore M. Kamenecka, Laura A. Solt

**Affiliations:** 1 Department of Immunology and Microbiology, The Scripps Research Institute, Jupiter, FL 33458 USA; 2 Department of Integrative Structural and Computational Biology, The Scripps Research Institute, Jupiter, FL 33458 USA; 3 Skaggs Graduate School of Chemical and Biological Sciences, The Scripps Research Institute, Jupiter, FL 33458 USA; 4 Department of Molecular Medicine, The Scripps Research Institute, Jupiter, FL 33458 USA; 5 Present Address: Mater Research Institute, The University of Queensland, Woolloongabba, QLD 4102 Australia; 6 Present Address: Gale and Ira Drukier Institute for Children’s Health, Department of Pediatrics, Weill Cornell Medicine, New York, NY 10021 USA

**Keywords:** Target validation, Immunology, Adaptive immunity, Autoimmunity, Inflammation

## Abstract

Full development of IL-17 producing CD4^+^ T helper cells (T_H_17 cells) requires the transcriptional activity of both orphan nuclear receptors RORα and RORγt. However, RORα is considered functionally redundant to RORγt; therefore, the function and therapeutic value of RORα in T_H_17 cells is unclear. Here, using mouse models of autoimmune and chronic inflammation, we show that expression of RORα is required for T_H_17 cell pathogenicity. T-cell-specific deletion of RORα reduces the development of experimental autoimmune encephalomyelitis (EAE) and colitis. Reduced inflammation is associated with decreased T_H_17 cell development, lower expression of tissue-homing chemokine receptors and integrins, and increased frequencies of Foxp3^+^ T regulatory cells. Importantly, inhibition of RORα with a selective small molecule antagonist mostly phenocopies our genetic data, showing potent suppression of the in vivo development of both chronic/progressive and relapsing/remitting EAE, but with no effect on overall thymic cellularity. Furthermore, use of the RORα antagonist effectively inhibits human T_H_17 cell differentiation and memory cytokine secretion. Together, these data suggest that RORα functions independent of RORγt in programming T_H_17 pathogenicity and identifies RORα as a safer and more selective therapeutic target for the treatment of T_H_17-mediated autoimmunity.

## Abstract (web summary)

Unlike RORα, which has been thought to be somewhat redundant, RORγt has been well characterized in its function and contribution to the development of Th17 cells. Here the authors show that RORα is important in Th17 differentiation and that RORα deletion or a small molecule inhibitor of RORα can reduce disease in EAE and colitis mouse models.

## Introduction

T helper 17 (T_H_17) cells are an important lineage of CD4^+^ T effector (Teff) cells that survey mucosal barriers, secrete IL-17-family cytokines, and guard against extracellular bacteria and fungi at mucosal surfaces. Patients with defects in the T_H_17 pathway, including autosomal dominant Hyper-IgE syndrome (AD-HIES), suffer from recurrent infections to mucocutaneous fungal and bacterial infections, such as *Candida albicans* and *Staphylococcus aureus*^1–3^. In contrast, over-exuberant T_H_17 responses have been implicated in many human autoimmune diseases, including multiple sclerosis, rheumatoid arthritis, and inflammatory bowel disease (IBD)^4^. Genome-wide association studies indicate SNPs in multiple genes associated with the T_H_17 axis as susceptibility factors in autoimmunity^5,6^. As a consequence, a large effort has been put forth to target this pathway for the treatment of T_H_17-mediated diseases, and in particular to identify pharmacologically accessible mechanisms that block pathogenic, but not protective, T_H_17 cell functions.

The nuclear receptor (NR) retinoic acid receptor-related orphan receptor gamma t (RORγt; NR1F3) is considered the lineage defining transcription factor of T_H_17 cells^7^. Since its discovery over a decade ago, a significant amount of work has been invested into understanding RORγt’s function in T_H_17 cells, and to translate this understanding into new treatments for autoimmune and inflammatory syndromes. While RORγt is critical for T_H_17 cell development, IL-17A is not completely abolished in RORγt-deficient mice; its close family member, RORα (NR1F1), is also required for full T_H_17 cell development^8^. Thus, deletion of both receptors completely abolishes T_H_17 cell development^8^. Despite this, RORα is considered redundant to RORγt and as such the functions of RORα in protective and pathogenic T_H_17 cell responses have not been systematically explored.

Here we show that RORα is a critical factor required for the development of autoimmunity and chronic inflammatory responses using mouse models of multiple sclerosis and colitis. Importantly, as a member of the nuclear receptor superfamily, RORα is a ligand-regulated transcription factor and targeting of this receptor in vivo not only inhibits the development of autoimmunity, it effectively suppresses active inflammation. These effects extend to human T_H_17 cells, both during differentiation and in the regulation of pro-inflammatory cytokine expression in lineage-committed T_H_17 cell memory subsets. Unlike pharmacologic inhibition of RORγt, we show that RORα inhibition suppresses pro-inflammatory T_H_17-responses while inducing immunoregulatory function, while leaving thymic T cell development intact. Collectively, these data suggest that RORα is a non-redundant factor that is required for pathogenic T_H_17 cell function and represents an attractive alternative for treating T_H_17-associated inflammatory diseases in humans.

## Results

### RORα expression is sufficient to drive T_H_17 signature genes

**Fig. 1:**
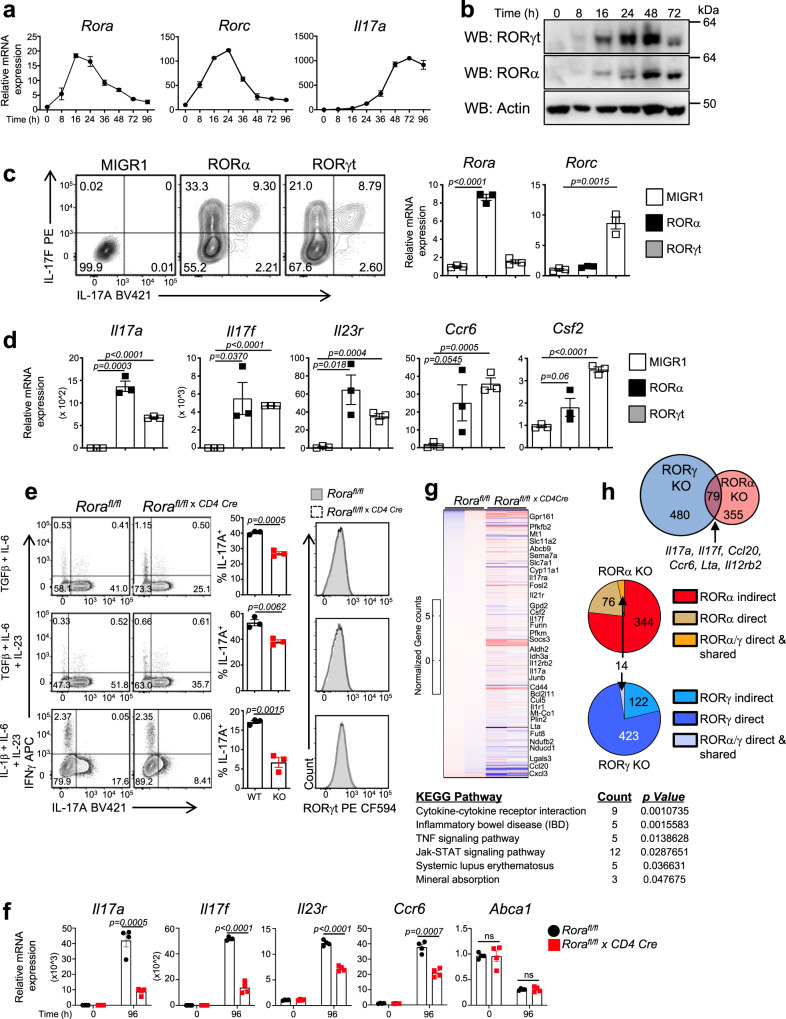
RORα is expressed and required for full T_H_17 cell differentiation. **a** Quantitative real-time PCR (qRT-PCR) analysis of RORα (*Rora*), RORγt (*Rorc*), and *Il17a* expression during T_H_17 cell development. β-actin was used as the internal control. **b** Western blot demonstrating protein expression of RORγt and RORα during T_H_17 cell development. Uncropped blots in Source Data. **c** FACS analysis of IL-17A and IL-17F expression in T cells transduced with empty vector (MIGR1), RORα, or RORγt. Cells were gated on live, GFP^+^ cells. qRT-PCR analysis of RORα and RORγt expression from sorted GFP^+^ cells (right) (**d**) qRT-PCR of sorted GFP^+^ cells from Panel c. β-actin was used as the internal control. **e** FACS analysis of IL-17A vs IFNγ expression (left panels) from T_H_17 cultures derived from *Rora*^*fl/fl*^ (WT) and *Rora*^*fl/fl* *×* *CD4 Cre*^ (KO) mice. Graphs (middle panels) indicate percent IL-17A^+^ cells in the cultures. FACS analysis (right panels) of RORγt expression from a representative culture condition. **f** qRT-PCR of T_H_17-mediated cytokines in T_H_17 cell cultures from WT and KO mice. β-actin was used as the internal control. **g** Heat map of differentially expressed genes between WT and KO T_H_17 cells. KEGG pathway analysis of genes differentially expressed between WT and KO T_H_17 cells (FDR < 0.1). **h** (Top) Venn diagram depicting the numbers of unique and shared genes differentially regulated in WT/*Rora*^*fl/fl* *×* *CD4 Cre*^ and WT/RORγ^−/−^ T_H_17 cells. Middle diagram shows the numbers of genes that are indirect or direct RORα target genes in WT/*Rora*^*fl/fl* *×* *CD4 Cre*^ T_H_17 cells. Bottom diagram shows similar data for WT/RORγ^−/−^ T_H_17 cells. A small number of direct target genes are shared between the two receptors (FDR < 0.1). Data are presented as mean values ± s.e.m. (*n* = 3). Student’s two-tailed *t* tests were performed for statistical analysis. ns not significant (*p* > 0.05). Source data are provided as a Source Data file.

While RORα has been reported to be expressed in T_H_17 cells, we wanted to establish its kinetic expression pattern during T_H_17 cell development^8^. Naïve CD4^+^ T cells were differentiated under T_H_17 polarizing conditions (TGFβ + IL-6) to assess the mRNA and protein expression of RORα versus RORγt. Quantitative real-time polymerase chain reaction (qRT-PCR) analysis demonstrated that RORα (*Rora*) was upregulated at similar time points as RORγt (*Rorc*) driving *Il17a* expression^7^ (Fig. 1a). This data is consistent with microarray analysis demonstrating that *Rora* is upregulated in both pathogenic and non-pathogenic T_H_17 cells to levels equal to or greater than *Rorc*^9^. Western blot analysis confirmed that RORα was expressed at the protein level (Fig. 1b).

To determine whether RORα alone was sufficient to drive IL-17A and T_H_17-specific gene expression, we retrovirally overexpressed empty vector (MIGR1), RORα, or RORγt under T_H_ neutral conditions (αIL-4 + αIFNγ, no cytokines) in stimulated naïve CD4^+^ T cells to assess IL-17A/F expression by flow cytometry. Neutral conditions were used to not confound results since TGFβ and IL-6 upregulate both RORs^8,10,11^. RORα was able to induce IL-17A expression, as was RORγt (Fig. 1c). GFP^+^ cells were sorted to perform qRT-PCR to compare RORα vs. RORγt’s ability to drive gene expression. RORα induced the expression of *Il17a*, *Il17f*, *Il23r*, *Ccr6*, and *Csf2*, all genes that are considered “core” RORγt target genes^12^ (Fig. 1d). These data demonstrate that RORα is capable of driving the T_H_17 cell developmental program.

Previous studies assessing RORα’s function in the immune system utilized the *Staggerer* (*Rora*^*sg/sg*^) mouse, a naturally occurring mutant mouse strain that renders RORα inactive^13^. To more specifically assess the role of RORα in mature CD4^+^ T cell responses, we developed RORα “floxed” mice and bred them with *Cd4* Cre mice to delete RORα from T cells (Supplementary Fig. 1A). Differentiation of naive CD4^+^ T cells under non-pathogenic and pathogenic T_H_17 inducing conditions [TGFβ + IL-6 (*top panels*); TGFβ + IL-6 + IL-23 (*middl*e *panels*); IL-6 + IL-1β + IL-23 (bottom panels)] from *Rora*^*fl/fl*^ and *Rora*^*fl/fl x CD4 Cre*^ mice indicated that despite equal amount of RORγt expression between WT and KO conditions, cells lacking RORα were unable to express IL-17A at similar levels as RORα sufficient cells (Fig. 1e). Consistent with previous reports, RORγt-deficient cells had a decreased ability to produce IL-17A^14^ indicating that combined expression of both RORs is required for full IL-17A expression (Supplementary Fig. 1B). We observed no difference in the development of naïve CD4^+^ T cells into T_H_0, T_H_1, T_H_2, inducible T regulatory (iTregs) or regulatory T (Tr1) cells from *Rora*^*fl/fl*^ and *Rora*^*fl/fl x CD4 Cre*^ mice (Supplementary Fig. 1C). qRT-PCR analysis of T_H_17 cells cultured under non-pathogenic and pathogenic T_H_17 inducing conditions (see Fig. 1e for conditions) demonstrated that RORα deficiency reduced T_H_17-specific gene expression, including *Il17a*, *Il17f*, *Il23r*, and *Ccr6* whereas no difference was observed in the expression of *Abca1* (Fig. 1f and Supplementary Fig. 2A, B).

To gain a more global understanding of how RORα affects T_H_17 cell development, we performed RNA-sequencing on T_H_17 cells from *Rora*^*fl/fl*^ and *Rora*^*fl/fl* *×* *CD4 Cre*^ mice (Fig. 1g). KEGG pathway analysis indicated RORα was involved in regulation of cytokine–cytokine receptor interactions, inflammatory bowel disease, TNF signaling pathways, and Jak-STAT signaling pathways. We compared our data to RORγ-deficient T_H_17 cells and found that RORα had a smaller gene signature than RORγt^12^, with only 79 genes in common between the two receptors in T_H_17 cells, including “core” RORγt target genes^12^ (*Il17a, Ccr6, Ccl20*, etc.) suggesting RORα and RORγt shared regulation of these genes, which is consistent with the little that is known about RORα’s role in T_H_17 cells^8^ (Fig. 1h). To begin to address RORα-and RORγt-direct vs. indirect target genes, we compared gene expression in RORα- and RORγt-deficient T_H_17 cells relative to RORα vs. RORγt cistromes from liver and T_H_17 cells, respectively^12,15^. The RORα liver cistrome will still give some indication of RORα direct target genes in tissues. Using this approach, we identified a small number of shared RORα/γt-direct target genes, including *Furin*, *Il17ra*, and *Il1r1*. While our analysis indicated a large number of RORα indirect target genes, including *Il17a, Ccl20*, *Socs3*, and *Ccr6*, this may be a consequence of probing the RORα cistrome in a tissue other than T cells. However, a number of interesting direct RORα (only) target genes were uncovered, including those located in the mitochondrial membrane, *Slc11a2*, *Idh3a*, *Gpd2*, and *Ndufb2*, suggesting RORα may play a role in the regulation of mitochondrial metabolism. T_H_17 pathogenicity has been shown to be regulated by high rates of cellular metabolism and changes in glycolysis, OXPHOS, fatty acid or glutamine metabolism can prevent T_H_17-mediated autoimmune disease^16–22^. Given most of these genes were downregulated, decreased OXPHOS could be a contributing factor to the decreased T_H_17 responses in vitro. Interestingly, *Fosl2*, previously identified as a key T_H_17 regulator^12^, also appears to be directly regulated by RORα. Given it acts as a repressor, regulation of *Fosl2* expression could also account for the decreased T_H_17 responses observed in RORα-deficient T_H_17 cells. These data indicate that RORα may regulate genes in diverse categories (i.e. ion transport, NADH metabolic processes, cell migration, etc.) that drive its function regulating cellular T_H_17 responses.

### RORα is required for the development of chronic inflammatory diseases

**Fig. 2:**
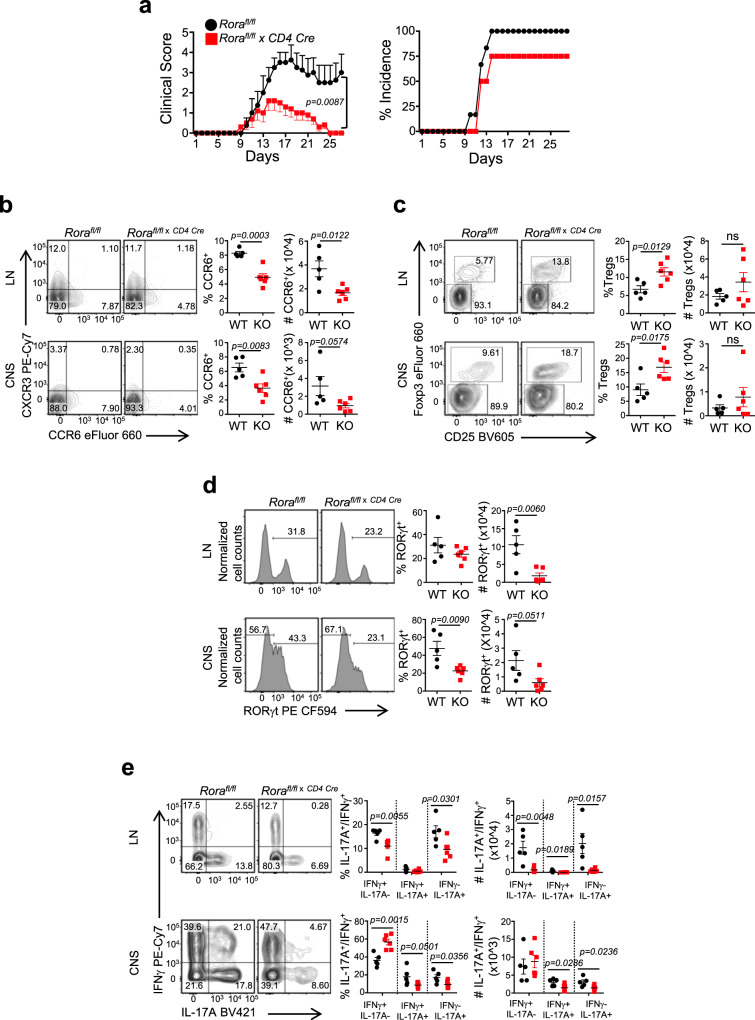
T-cell-specific loss of RORα attenuates the severity of EAE. **a** Clinical EAE scores (left) and disease incidence (right) from WT and KO mice subjected to MOG-induced EAE. Representative FACS plots and graphs (right) summarizing the frequency of **b** CCR6^+^ and **c** CD25^+^Foxp3^+^ T cells in the LN and CNS of WT vs. KO mice at the peak of disease. FACS analysis and graphs depicting the frequency and cell numbers of **d** RORγt expression and **e** IL-17A^+^IFNγ^−^, IL-17A^+^IFNγ^+^, and IL-17A^−^IFNγ^+^ cells in the LN and CNS of WT and KO mice at the peak of disease. Cells were gated on live, CD45^+^CD3^+^CD4^+^CD44^+^ cells. Each symbol represents an individual mouse [(WT, *n* = 5; αKO, *n* = 6) for panels **a**–**e**]. Data are presented as mean values ± s.e.m. Data are representative of two separate independent experiments generating similar results. Mann–Whitney *U* test for **a**, Student’s two-tailed *t* tests were performed for statistical analysis, **b**–**e**. Source data are provided as a Source Data file.

Recently, polymorphisms in *RORA* have been identified and associated with an increased risk for multiple sclerosis (MS)^23^. While found in non-coding regions, these SNPs were predicted to affect the affinity of transcription factor binding in regulatory regions of the *RORA* gene^23^. To establish how T-cell specific loss of RORα affected autoimmune disease development, we immunized *Rora*^*fl/fl*^ and *Rora*^*fl/fl* *×* *CD4 Cre*^ mice with MOG_35–__55_ peptide/complete freund’s adjuvant (CFA) plus pertussis toxin to induce experimental autoimmune encephalomyelitis (EAE), a commonly used T_H_17-dependent mouse model of chronic-progressive MS^24,25^. While the incidence of disease between the two groups was not significantly different, *Rora*^*fl/fl* *×* *CD4 Cre*^ mice displayed attenuated disease severity relative to *Rora*^*fl/fl*^ mice (Fig. 2a). Analysis of CD4^+^ T cells from mice killed at the peak of disease (~Day 15) demonstrated a decreased percentage and number of CCR6^+^ cells in the draining lymph nodes (LNs) and central nervous system (CNS) in the *Rora*^*fl/fl* *×* *CD4 Cre*^ mice (Fig. 2b and Supplementary Fig. 3). CCR6 is a chemokine receptor that permits T_H_17 cell migration into inflamed peripheral tissues, including the CNS and gut^26^ and is thought to be a RORγt target gene^12^. Genetic deletion or inhibition of CCR6 function has been shown to significantly reduce the severity of EAE^27^. Interestingly, there appeared to be an increase in the percent of CD25^+^Foxp3^+^ cells in the LNs and CNS of *Rora*^*fl/fl* *×* *CD4 Cre*^ mice compared to *Rora*^*fl/fl*^ control mice (Fig. 2c). This correlated with the decreased percent and number of RORγt^+^ cells in LNs and CNS (Fig. 2d). In line with this, there were significantly less IL-17A^+^ cells, and importantly, significantly less IL-17A^+^IFNγ^+^ cells in *Rora*^*fl/fl* *×* *CD4 Cre*^ mice compared to *Rora*^*fl/fl*^ control mice (Fig. 2e). IL-17A^+^IFNγ^+^ cells have been linked to pathogenicity in several models of chronic disease, including EAE/MS^28–31^. These data suggest that RORα plays an important, non-redundant role to RORγt in the development of EAE.

**Fig. 3:**
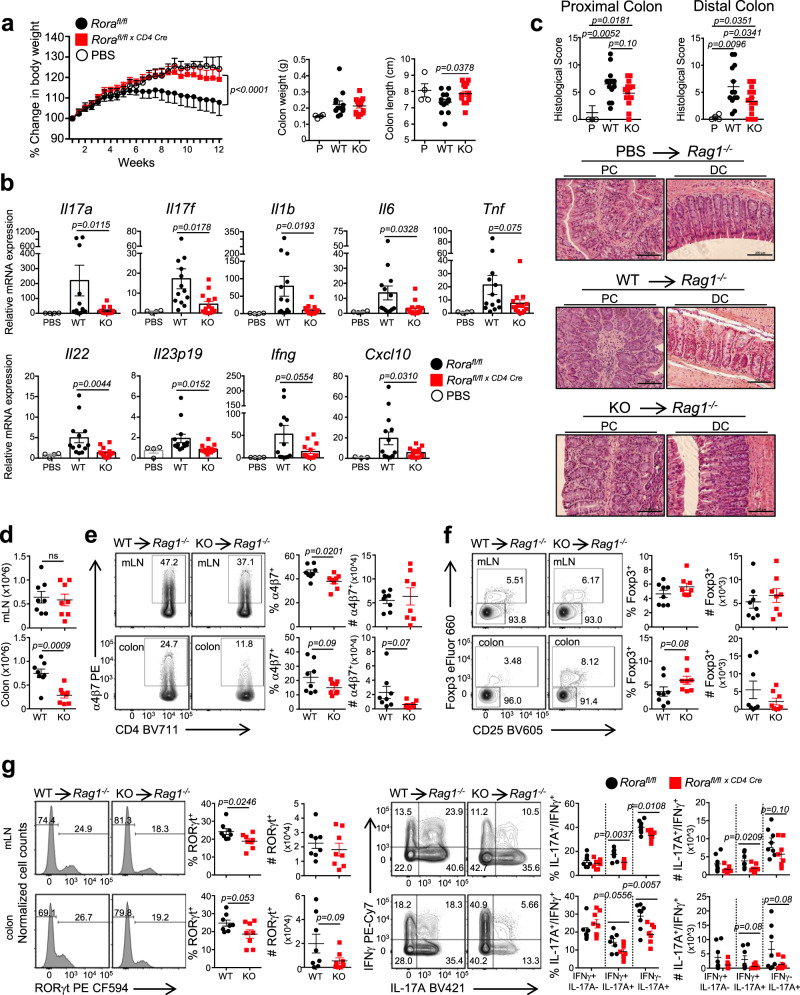
RORα-deficient T cells protect from the development of colitis. **a** Percent change in body weight, colon weights, and colon lengths of *Rag1*^*−/−*^ recipient mice over 12 weeks post-adoptive transfer of *Rora*^*fl/fl*^ (WT), *Rora*^*fl/fl* *×* *CD4 Cre*^ (KO), or control (no cells, PBS) T cells. **b** qRT-PCR analysis of cytokines expressed in the proximal colon from *Rag1*^*−/−*^ mice receiving PBS, *Rora*^*fl/fl*^ (WT), or *Rora*^*fl/fl* *×* *CD4 Cre*^ (KO) CD4^+^ T cells. 18 s was used as the internal control. **c** Histology scores from proximal and distal colons from *Rag1*^*−/−*^ mice receiving PBS, *Rora*^*fl/fl*^ (WT), or *Rora*^*fl/fl* *×* *CD4 Cre*^ (KO) CD4^+^ T cells (tops graphs). Representative hematoxylin and eosin stained sections from proximal and distal colons from the same mice 12 weeks post T-cell transfer (×20 magnification, scale bars 200 μm). [*n* = 4 (PBS), *n* = 13 (WT), and *n* = 14 (KO) for panels **a**–**c**] **d** Cell counts from mesenteric lymph nodes (mLN) and colons of mice receiving WT and KO T cells at 6 weeks post-transfer. Representative FACS plots and graphs (right) summarizing the frequency and cell numbers of **e** α4β7^+^ and **f** CD25^+^Foxp3^+^ T cells in the mLN and colons of WT vs KO mice at 6 weeks post-transfer. **g** FACS analysis and graphs depicting the frequency and cell numbers of RORγt expression (left) and IL-17A^+^IFNγ^−^, IL-17A^+^IFNγ^+^, and IL-17A^−^IFNγ^+^ cells in the mLN and colons of WT and KO mice at 6 weeks post-transfer. Cells were gated on live, CD45^+^CD3^+^CD4^+^CD44^+^ cells. Each symbol represents an individual mouse [*n* = 8 (WT), *n* = 8 (KO) for panels **e**–**g**]. Data are presented as mean values ± s.e.m. Data are representative of two combined independent experiments. Mann–Whitney *U* test for **a**, Student’s two-tailed *t* tests were performed for statistical analysis, **b**–**g**. Source data are provided as a Source Data file.

We next assessed whether loss of RORα affects the development of colitis, another chronic inflammatory disorder associated with increased T_H_17 differentiation and function^29,32^. We sorted naïve CD4^+^ T cells from *Rora*^*fl/fl*^ and *Rora*^*fl/fl x CD4 Cre*^ mice and transferred them (i.p.) into *Rag1*^*−/−*^ recipients and monitored their weight over time. PBS/sham injected mice were used as a control. Mice receiving *Rora*^*fl/fl* *×* *CD4 Cre*^ T cells developed minimal signs of disease (weight loss, diarrhea, changes in colon length), unlike mice receiving *Rora*^*fl/fl*^ naïve CD4^+^ T cells (Fig. 3a). Pro-inflammatory cytokine gene expression from proximal colons, including *Il17a*, *Il17f*, *Il1b*, *Il6*, *Tnf*, *Ifng*, *Il22*, and *Il23p19*, was also significantly decreased in mice receiving *Rora*^*fl/fl* *×* *CD4 Cre*^ T cells compared to *Rora*^*fl/fl*^ recipients (Fig. 3b). We also observed decreased expression of *Cxcl10*, a gene that is activated downstream of IL-17 and IL-22 signaling, which is consistent with the overall decreased T_H_17 proinflammatory profile observed in vivo. As expected, histological analysis revealed that mice receiving *Rora*^*fl/fl*^ T cells developed more severe colitis, evidenced by the increased cellular infiltration, broadening of crypts, and epithelial hyperplasia (Fig. 3c). In contrast, mice receiving *Rora*^*fl/fl* *×* *CD4 Cre*^ T cells showed mild signs of inflammation in the proximal and distal colons. Analysis of mice at 6 weeks post-transfer indicated there were less *Rora*^*fl/fl* *×* *CD4 Cre*^ cells infiltrating the colon, likely due to *Rora*^*fl/fl* *×* *CD4 Cre*^ cells expressing significantly less α4β7, an integrin that mediates lymphocyte extravasion to the intestinal lamina propria^33^ (Fig. 3d, e). Similar to what was observed in the EAE model, *Rag1*^*−/−*^ recipients receiving *Rora*^*fl/fl* *×* *CD4 Cre*^ cells showed an increase in the percent of CD25^+^Foxp3^+^ cells in the mesenteric LNs (mLNs) and colons compared to mice receiving *Rora*^*fl/fl*^ cells (Fig. 3f). Mice receiving *Rora*^*fl/fl* *×* *CD4 Cre*^ T cells also had significantly less RORγt, IL-17A^+^, and IL-17A^+^IFNγ^+^ cells (Fig. 3g). Populations of T cells simultaneously expressing both cytokines have been described in intestinal biopsies of IBD patients^34,35^. Collectively, our genetic data suggests that RORα plays a significant role in the development of T_H_17 cells, pathogenicity, and T_H_17-mediated pro-inflammatory diseases.

### RORα controls T_H_17 cell responses through a cell intrinsic manner in vivo

**Fig. 4:**
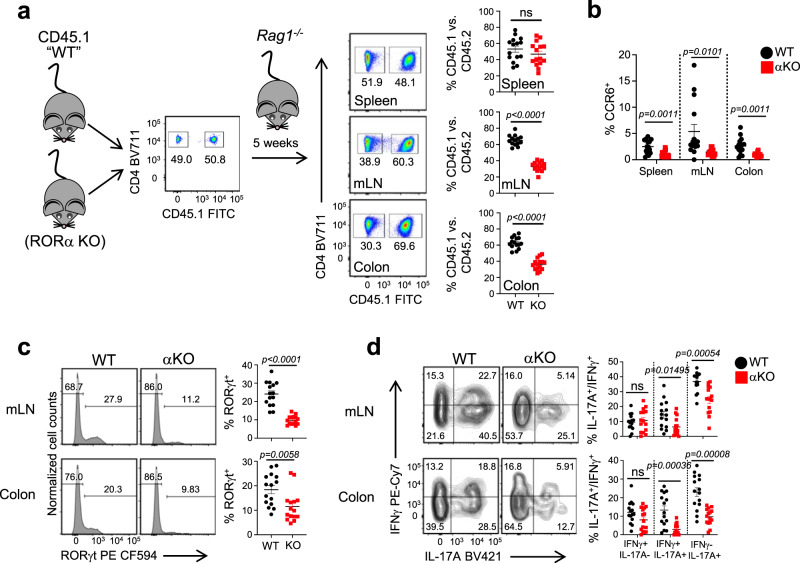
Defects in RORα-deficient T cells in colitis is cell intrinsic. **a** Schematic of experimental set up. Naïve CD4^+^ T cell were sorted from CD45.1 (WT) and RORα T cell deficient (αKO) mice and transferred into *Rag1*^*−/−*^ recipient mice. Representative FACS plot from one experiment (*left panel*). Right panels; representative FACS plots and graphs demonstrating the frequency of WT vs. αKO T cells present in the spleens, mLNs, and colons of *Rag1*^*−/−*^ recipient mice 5 weeks post-transfer. **b** Graphs summarizing the frequency of CCR6^+^ cells in each tissue. FACS analysis and graphs depicting the frequency of **c** RORγt expression and **d** IL-17A^+^IFNγ^−^, IL-17A^+^IFNγ^+^, and IL-17A^−^IFNγ^+^ cells in the mLN and colons of WT and αKO mice at 5 weeks post-transfer. Cells were gated on live, CD3^+^CD44^+^CD4^+^ and CC45^+^ or CD45^−^ cells. Each symbol represents an individual mouse [*n* = 15 (WT), *n* = 15 (αKO) for panels **a**–**d**]. Data are presented as mean values ± s.e.m. Data are representative of two combined independent experiments. Student’s two-tailed *t* tests were performed for statistical analysis. Source data are provided as a Source Data file.

To address whether RORα affects T_H_17 cell function through cell-intrinsic or cell-extrinsic mechanisms in vivo, we took advantage of a co-transfer model. We transferred equal numbers of congenic CD45.1^+^ (WT) or CD45.2^+^ RORα KO (KO) naïve CD4^+^ T cells (i.p.) into *Rag1*^*−/−*^ recipients (Fig. 4a). After 5 weeks of expansion, there was no observable difference in the ratio between WT and KO T cells in the spleen. However, there was a significant difference in the ratio of WT to KO T cells in the mLNs and colon lamina propria (LP) with WT markedly outcompeting KO T cells (Fig. 4a). Despite this phenotype observed in the spleen, we observed a decreased percentage of CCR6^+^ cells in all KO T cells relative to WT in all tissues (spleen, mLNs, and colon LP; Fig. 4b). RORα KO T cells also expressed significantly decreased amounts of RORγt^+^ cells as well as IL-17A^+^ and IL-17A^+^IFNγ^+^ cells in mLN and colon LP relative to WT T cells (Fig. 4c, d). These results demonstrate that RORα is required for T cell accumulation in the intestine and the downstream T_H_17 cell pro-inflammatory response. Furthermore, it cannot be rescued by the presence of RORα-sufficient T cells. Therefore, RORα activity in T_H_17 cells is required for their full effector function in vivo through a cell-intrinsic manner.

### A RORα-selective small molecule regulates T_H_17 cell development

**Fig. 5:**
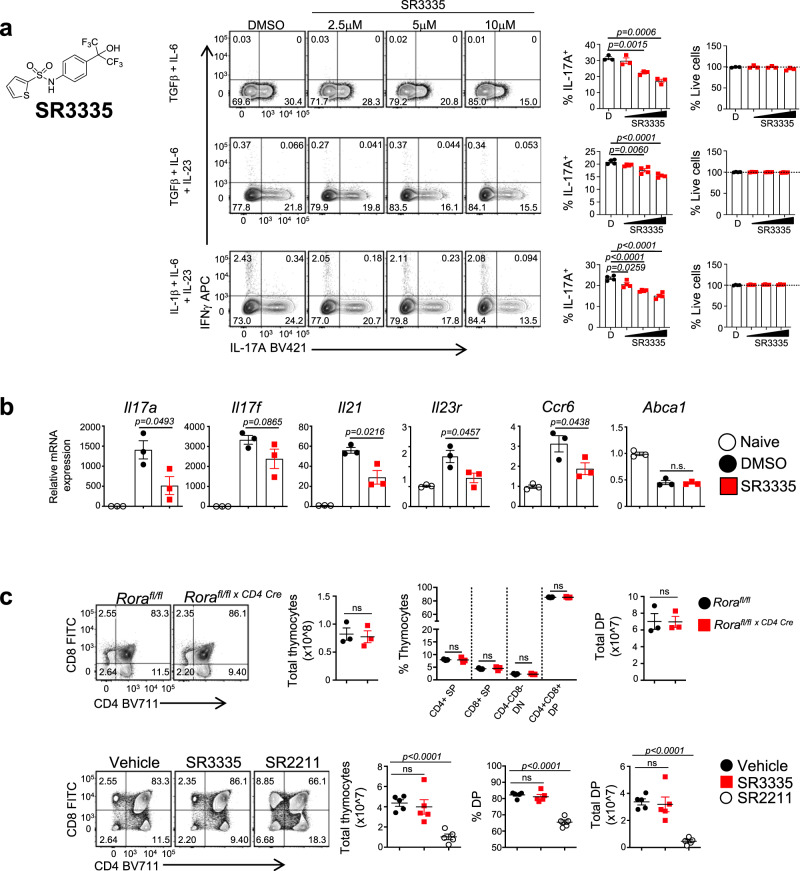
RORα-specific small molecule suppresses T_H_17-cell development. **a** Mouse naïve CD4^+^ T cells were differentiated under three different T_H_17 polarizing conditions (non-pathogenic and pathogenic) and treated with vehicle (DMSO) or SR3335. IL-17A and IFNγ expression were analyzed by flow cytometry. Graphs indicate percent IL-17A^+^ cells and frequency of live cells in cultures with compound treatment. **b** qRT-PCR of T_H_17-mediated cytokines in cells treated with vehicle (DMSO) or SR3335 (5μM). Analysis was performed at 96 h post T-cell activation and compared to naïve CD4^+^ T cells. β-actin was used as the internal control. *n* = 3 technical replicates. **c** FACS analysis of thymocytes from 8-week-old *Rora*^*fl/fl*^ (WT) or *Rora*^*fl/fl* *×* *CD4 Cre*^ (KO) mice (*top panels*). Graphs demonstrate the total thymocyte number, frequency of thymocytes per subset, and total double positive (DP) cell number. (SP single positive, DP double positive, DN double negative; *n* = 3/group). (bottom panels) FACS analysis of mice treated with vehicle, SR2211, or SR3335 for 72 h. Graphs depict total thymocyte number, percent of double positive cells, and number of double positive cells in each group. Data are presented as mean values ± s.e.m. Data shown are representative of two separate, independent experiments (*n* = 5/group). Student’s two-tailed *t* tests were performed for statistical analysis, ns not significant (*p* > *0.05*). Source data are provided as a Source Data file.

We previously identified and characterized SR3335, a proof-of-concept RORα-selective inverse agonist^36^. To date, SR3335 is the only RORα inverse agonist in the primary literature that has been demonstrated to bind RORα and modulate its activity^36^. When tested against all 48 human nuclear receptors, SR3335 exhibited modest activity at LXRβ and PXR, but this activity was negligible compared to full LXR and PXR agonists T0901317^37^ and Rifampicin^38^, respectively (Supplementary Fig. 4A–C). To determine whether pharmacological modulation of RORα’s activity can affect IL-17A expression, naïve CD4^+^ T cells were differentiated under several T_H_17 conditions (non-pathogenic and pathogenic) in the presence of SR3335 or vehicle control. SR3335 dose dependently suppressed IL-17A expression without affecting cell viability (Fig. 5a). This effect was specific to RORα and T_H_17 cells as the effect was lost when tested in *Rora*^*fl/fl* *×* *CD4 Cre*^ T_H_17 cells nor did SR3335 affect the development of T_H_1 or iTregs cells (Supplementary Fig. 4D, E). Gene expression analysis indicated that SR3335 inhibited the expression of *Il17a*, *Il17f*, *Il21*, *Il23r*, and *Ccr6* without affecting the expression of the LXR target gene *Abca1* (Fig. 5b). Since loss of RORγ and many recently developed RORγ modulators induce thymic apoptosis, we wanted to determine if genetic deletion and/or targeting RORα induced similar effects. Unlike RORγ^39^, T-cell-specific deletion of RORα had no effect on T cell development in the thymus (Fig. 5c, upper panels). To determine if SR3335 had any effects on thymic apoptosis, we treated C57BL/6 mice for 3 days with SR3335 (30 mg/kg, b.i.d, i.p.), the RORγ-selective inverse agonist SR2211^40^ (20 mg/kg, b.i.d., i.p.), or vehicle controls. After 3 days, mice were killed to collect thymi to assess T cell development and total thymic cell numbers. Unlike mice treated with SR2211, which demonstrated decreased overall thymic cell number, percent of cells in the double positive stage, and total number of cells in the double positive stage, mice treated with SR3335 resembled vehicle control treated mice, indicating SR3335 had little effect on the thymus (Fig. 3c, lower panels). These data indicate that RORα-selective inverse agonists can effectively target the T_H_17 pathway in vitro but do not appear to carry the associated risks of thymic apoptosis that RORγ inverse agonists do.

### SR3335 inhibits the development of autoimmunity

**Fig. 6:**
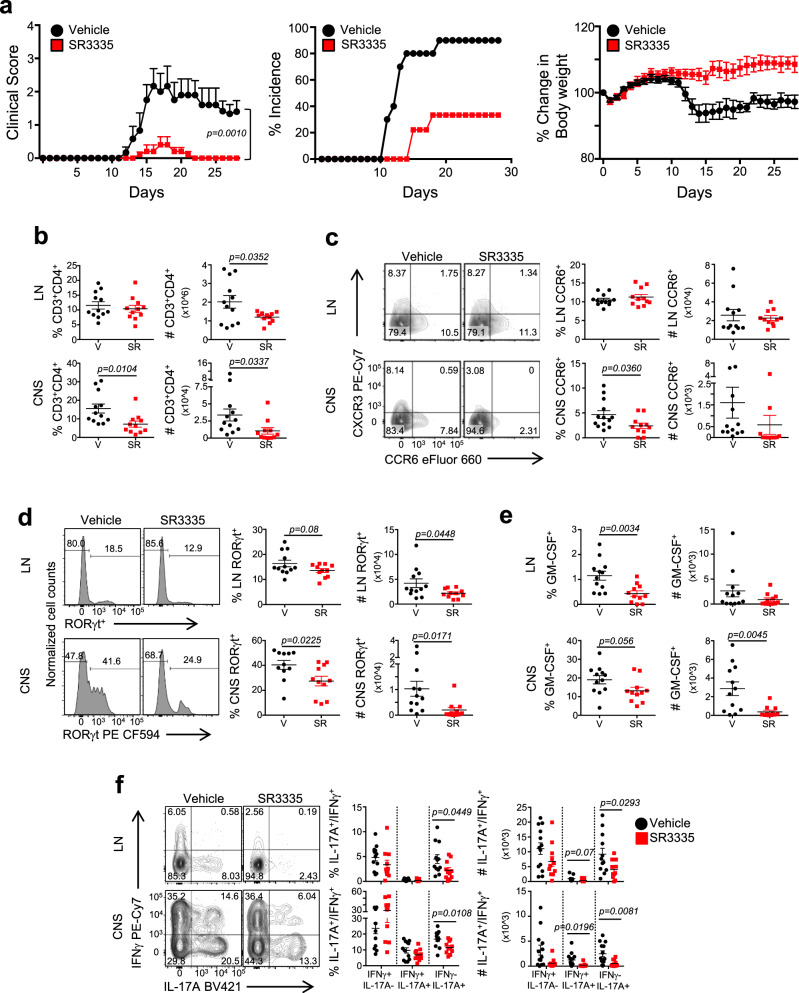
SR3335 suppresses the development and severity of chronic EAE. **a** Clinical EAE scores (left) from mice subjected to MOG-induced EAE and treated with Vehicle (10/10/80 formulation of DMSO/Tween80/H_2_O) or SR3335 (i.p., 30 mg/kg, b.i.d.) for the duration of the experiment. Middle graph demonstrates the percent incidence of disease. Right graph demonstrates the percent change in body weight over time between the two groups. (Vehicle, *n* = 10; SR3335, *n* = 10) Data representative of three separate, independent experiments with similar results. **b** Frequencies and cell number of CD3^+^CD4^+^ cells in the draining lymph nodes (LN) and CNS of mice at peak of disease. **c** Representative FACS plots and graphs (right) summarizing the frequency and cell number of CCR6^+^ and T cells in the LNs and CNS of vehicle vs. drug treated mice at the peak of disease. **d** FACS analysis, frequencies, and cell counts of RORγt^+^ cells in LNs and CNS of mice at peak of disease. **e** Graphs depicting the frequencies and cell counts of CD4^+^GM-CSF^+^ cells in LN and CNS of mice at peak of disease. **f** FACS analysis, frequencies, and cell counts of IL-17A^+^IFNγ^−^, IL-17A^+^IFNγ^+^, and IL-17A^−^IFNγ^+^ cells in the LN and CNS of mice at the peak of disease. Cells were gated on live, CD45^+^CD3^+^CD4^+^CD44^+^ cells. [V = Vehicle (*n* = 12), SR = SR3335-treated mice (*n* = 11) for panels **b**–**f**]. Data are presented as mean values ± s.e.m. Data are representative of two combined independent experiments with similar results. Mann–Whitney *U* test for **a**, Student’s two-tailed *t* tests were performed for statistical analysis, **b**–**f**. Source data are provided as a Source Data file.

To establish whether SR3335 was effective at inhibiting the development of autoimmunity in vivo, we immunized C57BL/6 mice to induce EAE and treated them with SR3335 (i.p. 30 mg/kg, B.I.D.) or vehicle control following immunization for the duration of the experiment. Dosing was based on drug metabolism and pharmacokinetic experiments performed in-house (Supplementary Fig. 5A). Disease development and incidence was delayed and significantly reduced in SR3335-treated animals relative to vehicle controls (Fig. 6a). Weights of the animals indicated no overt toxicity of the drug. Analysis of CD4^+^ T cells at the peak of disease indicated a significantly decreased frequency of CD3^+^CD4^+^ T cells in the CNS whereas the numbers of cells were decreased in the LNs and the CNS (Fig. 6b). The decreased frequency and number of cells in the CNS was likely due to the decreased percent of CCR6^+^ cells in the CNS (Fig. 6c). SR3335 treatment led to a significant decrease in the frequency and number of RORγt^+^ cells in the LNs and CNS (Fig. 6d). This correlated with the decreased frequency and number of GM-CSF^+^ and IL-17A^+^ cells in the LNs and CNS (Fig. 6e, f). GM-CSF-expressing T_H_17 cells have been shown to be highly encephalitogenic^41,42^ (Fig. 6e). While there was only a very small difference in the frequency of IL-17A^+^IFNγ^+^ cells in the SR3335-treated mice, the absolute numbers of these cells were significantly lower in the CNS of the drug-treated mice compared to vehicle controls (Fig. 6f). These data suggest that treatment with a RORα-selective ligand largely phenocopy’s the genetic data and is effective at inhibiting the development of autoimmunity.

### RORα-selective ligands are inhibitory during active inflammation

**Fig. 7:**
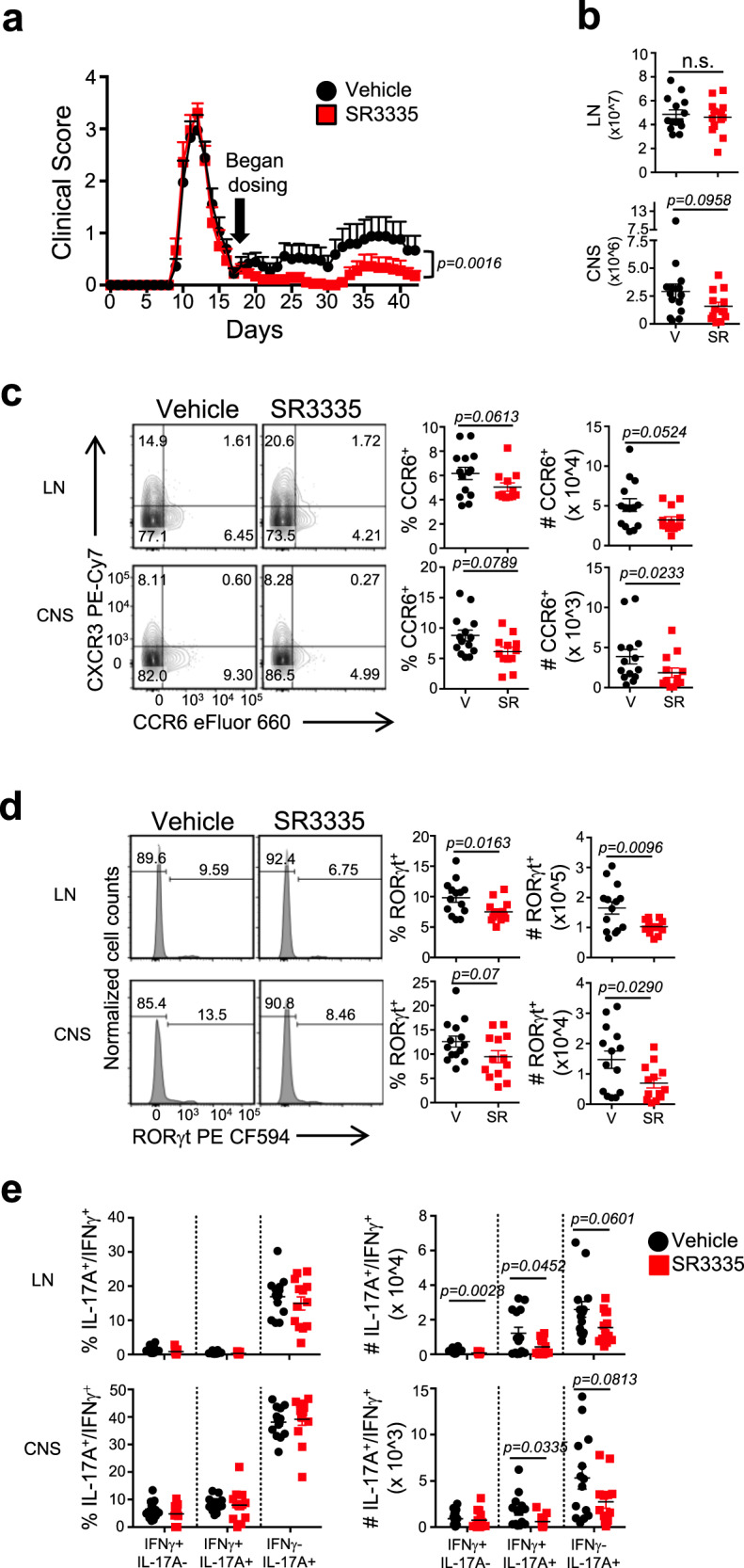
SR3335 is effective when used therapeutically in relapsing-remitting EAE. **a** Clinical EAE scores of mice from relapsing-remitting model of EAE treated with vehicle or SR3335 (i.p., 30 mg/kg, b.i.d.) starting on day 18 and continued for the duration of the experiment (*n* = 18/group). Data are representative of two separate experiments. **b** Cell counts from draining lymph nodes (LN) and CNS of mice at the termination of the experiment. FACS plots, frequencies and cell counts of **c** CCR6^+^ and **d** RORγt^+^ T cells in the LNs and CNS of vehicle and SR3335-treated mice. **e** Graphs representing the frequencies and cell counts of IL-17A^+^IFNγ^−^, IL-17A^+^IFNγ^+^, and IL-17A^−^IFNγ^+^ cells in the LNs and CNS of mice treated with vehicle and SR3335. [(*n* = 14, vehicle; *n* = 13, SR3335) for panels **c**–**e**]. Data are presented as mean values ± s.e.m. Data are representative of two combined independent experiments with similar results. Mann–Whitney *U* test for **a**, Student’s two-tailed *t* tests were performed for statistical analysis, **b**–**e**. Source data are provided as a Source Data file.

To evaluate whether SR3335 was effective during active inflammation we tested SR3335 in the PLP_139-151_-induced relapsing-remitting EAE (R-EAE) model. SR3335 (30 mg/kg, B.I.D.) or vehicle was administered once mice had recovered from the first wave of disease (day 18 post-immunization). SR3335 significantly reduced the relapse rate and severity of relapse compared to animals receiving vehicle (Fig. 7a). Similar to the chronic EAE model, SR3335 appeared to inhibit cellular infiltration into the CNS, indicated by the decreased number of cells in the CNS relative to the draining lymph nodes (Fig. 7b). This was likely due to the decreased expression of CCR6 on CD4^+^ T cells (Fig. 7c). Intracellular FACS analysis demonstrated a decreased frequency and number of RORγt^+^ cells in the LNs and CNS of the SR3335-treated mice relative to vehicle controls (Fig. 7d). While we did observe a small decrease in IL-17A expression in the LNs of these mice, we did not observe a difference in the frequency of RORγt^+^IL-17A expression in the CNS of vehicle versus SR3335-treated mice. However, there was a significant reduction of the number of IL-17A^+^ and pathogenic IL-17A^+^IFNγ^+^ cells in CNS compared to mice receiving vehicle control (Fig. 7e). These results offer proof-of-concept that pharmacological modulation of RORα activity post-disease onset (therapeutic mode) can suppress the progression of T_H_17-driven autoimmunity and chronic inflammatory disorders.

### SR3335 regulates memory T_H_17 and T_H_17.1 cells

**Fig. 8:**
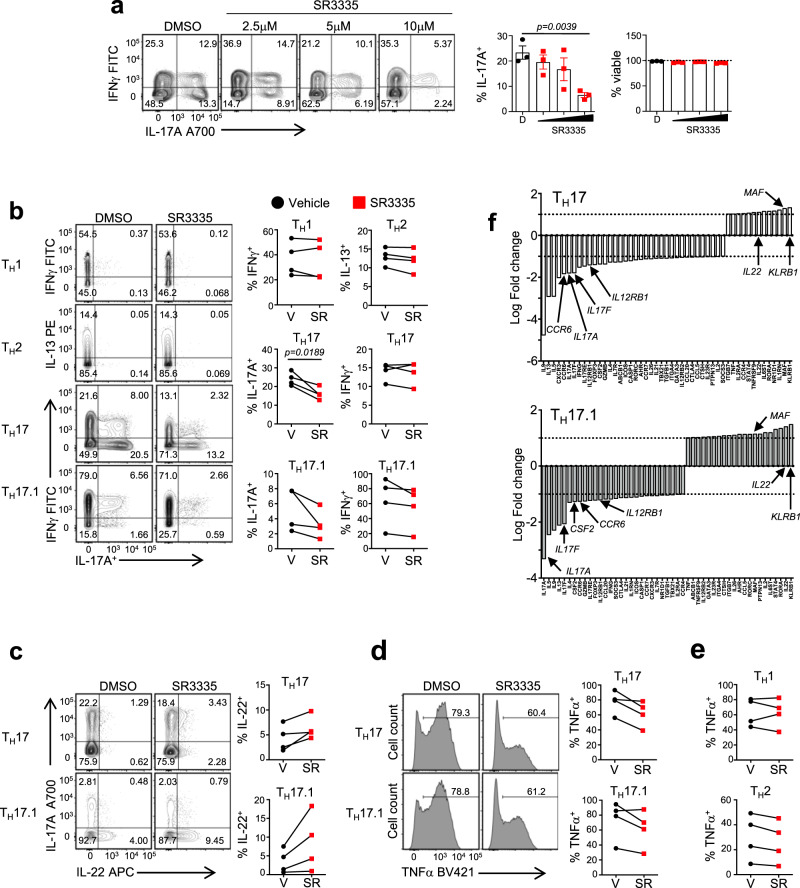
SR3335 modulates human memory T_H_17 and T_H_17.1 cells. **a** Naive human CD4^+^ T cells were differentiated under T_H_17 polarizing conditions (anti-CD3 + IL-23+IL-1β) and treated with vehicle (DMSO) or SR3335. IL-17A and IFNγ expression were analyzed from live, CD45RO^+^ cells by flow cytometry on Day 6. Graphs indicate percent IL-17A^+^ cells and frequency of live cells in cultures with compound treatment. **b** FACS sorted memory cell (CD4^+^CD25^−^CD45RO^+^) subsets from healthy adult donor peripheral blood were stimulated with PMA and ionomycin to determine cytokine production by flow cytometry after 6 days in culture in the presence of vehicle (V, DMSO) or SR3335 (SR, 5μM). Graphs depict percentage cytokine expression (IL-17A, IL-13, and IFNγ) in the cultures for each subset/sample. FACS analysis of T_H_17 and T_H_17.1 sorted memory cells to determine **c** IL-17A and IL-22 expression and **d** TNFα expression. **e** Graphs summarizing the percentage TNFα expression in sorted T_H_1 and T_H_2 memory cells treated with vehicle (V, DMSO) or SR3335 (SR, 5 μM) for 6 days from four different donors. **f** Nanostring data demonstrating the SR3335-mediated changes in gene expression in T_H_17 and T_H_17.1 cultures on day 6 from the combined data of three donors described in panel **e**. Data are presented as mean values ± s.e.m. Student’s two-tailed *t* tests were performed for statistical analysis. Source data are provided as a Source Data file.

It has been difficult to differentiate naïve human CD4^+^ T cells into T_H_17 cells that secrete a substantial amount of IL-17A. However, recent work has demonstrated that anti-CD3 stimulation in the presence of IL-23 and IL-1β, without anti-CD28, is sufficient to drive an abundant IL-17 response in human T cells^43^. To determine if SR3335 was effective at inhibiting human T_H_17 cell differentiation, we isolated human naïve CD4^+^ T cells from peripheral blood mononuclear cells (PBMCs) and differentiated them under conditions described by Revu et al.^43^. Similar to mouse T_H_17 cells, SR3335 dose dependently inhibited the development of human T_H_17 cells without affecting cell viability (Fig. 8a). To establish whether inhibition of RORα activity would affect T_H_17 cells derived from patients with IBD, specifically Ulcerative Colitis (UC), we differentiated naïve CD4^+^ T cells from PBMCs from UC patients vs. healthy, age-matched controls (HC) under conditions described in Fig. 8a. While PBMCs from UC patients consistently yielded greater amounts of IL-17A and IFNγ, SR3335 was still able to inhibit IL-17A in a dose dependent manner (Supplementary Fig. 5b).

IL-17A expressing memory cells (CD45RO^+^) are enriched within PBMCs and can be identified by their co-expression of CCR6 and CCR4^44^. However, CCR6^+^ human memory cells can be further subdivided based on expression of CXCR3, which describes a subset of cells that expresses both IL-17A and IFNγ upon ex-vivo stimulation^45^. These two populations of cells have been demonstrated to be stable and are referred to as T_H_17 (CCR6^+^CCR4^hi^CXCR3^lo^) and T_H_17.1 (CCR6^+^CCR4^lo^CXCR3^hi^)^46,47^. To determine whether SR3335 could affect IL-17A expression in human memory T_H_17 subsets, we sorted memory T helper cell subsets based on expression of CCR6, CCR4, and CXCR3 to isolate memory T_H_1, T_H_2, T_H_17, and T_H_17.1 populations. (All populations were CD4^+^CD45RO^+^CD25^−^CRTH2^−^CCR10^−^.) Sorted populations were cultured for 6 days in the presence of vehicle or SR3335, after which time cells were FACS analyzed to determine intracellular cytokine profiles. Consistent with the mouse in vitro cultures, SR3335 had little effect on the T_H_1 (CCR6^−^CCR4^lo^CXCR3^hi^) and T_H_2 (CCR6^−^CCR4^hi^CXCR3^lo^) cultures (Fig. 8b). However, SR3335 significantly inhibited the expression of IL-17A in the human memory T_H_17 cultures but did not quite reach significance in the T_H_17.1 cultures. Treatment with SR3335 also led to a small but reproducible increase in expression of IL-22 (Fig. 8c) while inhibiting TNFα expression in T_H_17 and T_H_17.1 populations, without affecting TNFα expression in T_H_1 or T_H_2 memory populations (Fig. 8d, e). This SR3335-mediated increase in IL-22 extends to mouse memory cells as culture of sorted mouse CCR6^+^ cells treated with SR3335 induced a similar response (Supplementary Fig. 5C). Gene expression analysis from these samples revealed that SR3335 inhibited expression of many “core” T_H_17 genes, including *IL17A*, *IL17F*, *CCR6*, and *IL12RB1*, which was consistent with much of the mouse data (Fig. 8f). Interestingly, SR3335 treatment also led to an increase in the transcription factor *MAF* in T_H_17 and T_H_17.1 populations. Collectively, these results indicate that RORα-selective inverse agonists can inhibit T_H_17 cell functions in lineage-committed human memory T cells as well as from patients with T_H_17 inflammatory diseases, like UC.

## Discussion

Here we used a combination of genetic, molecular biology, and pharmacological approaches to demonstrate that RORα is a major regulator of T_H_17 cell development, autoimmunity, and chronic inflammation. We demonstrated that in vitro, despite equal amounts of RORγt in cells, loss of RORα leads to a reduction in IL-17A expression, with no effect on the development of other T helper cell populations, including Foxp3^+^ T regulatory cells. Using mouse models of EAE and colitis we show that loss of RORα affected the expression of IL-17A, RORγt, as well as the frequency of CD25^+^Foxp3^+^ Tregs in vivo. Pharmacological studies demonstrated that use of a RORα-selective inverse agonist, SR3335, inhibited the development of mouse T_H_17 cells in vitro and in vivo, while leaving thymic T cell development intact, unlike RORγ modulators. Importantly, we determined that SR3335 effectively inhibits human T_H_17 cells as well.

Despite its clear effects and synergism with RORγt on T_H_17 cell development^8^, the functional role of RORα was never expanded upon. Importantly, the T-cell specific role of RORα or its effects in other models of chronic inflammation were never explored because RORα was considered “redundant” to RORγt. In nuclear receptor research, there are many receptor sub-families where one member is considered “dominant” and the other “redundant.” However, recent studies have started to tease apart the distinct signaling pathways for receptors in these sub-families demonstrating that they each possess a unique, requisite function in each cell type. For example, RXRα (NR2B1) and RXRβ (NR2B2) were recently shown to have distinct functions in retinoic acid signaling and neuronal differentiation^48^. Both LXRα (NR1H2) and LXRβ (NR1H3) were each found selectively associated with sequence motifs of specific transcription factors demonstrating LXRα- or LXRβ-selective gene regulation in macrophages^49^. Since redundancy suggests dispensable or unnecessary, our data indicates that RORα is a requisite factor for T_H_17 cell differentiation and the development of autoimmunity. Indeed, a recent publication has hinted at RORα’s function in T_H_17 cell pathogenicity in a *Rip2*-dependent fashion. In this report, *Rip2* negatively regulated the expression of RORα as *Rip2*^*−/−*^ CD4^+^ T cells expressed more RORα than WT CD4^+^ T cells^50^. The authors reported that RORα drove the expression of *Il1r1*, which they determined was RORα-dependent and was the basis for enhanced T_H_17 cell pathogenicity. While the increase in IL-1β signaling is consistent with increased pathogenicity observed in T_H_17 cells^51,52^, our data analysis has determined that *Il1r1* is regulated by both RORα and RORγt. Nevertheless, it still needs to be determined how and why Rip2 is regulating RORα expression in T_H_17 cells and what the biological significance of this is. Our genomics analysis did, however, identify some unique direct RORα target genes, including those that are important for mitochondrial metabolism (*Idh3a, Gpd2*, and *Ndufb2*), ion transport (*Slc11a2*), and other T_H_17 cell transcriptional regulators (*Fosl2*), underscoring the need to further probe the function of this transcription factor. What is interesting to note is that numerous genes, like *Ccr6*, *Il17a*, *Il23r*, did not appear to be direct RORα target genes. This is likely a function of the cell type used for ChIP-seq analysis and further analysis assessing RORα’s cistrome in T_H_17 cells is warranted to understand its full regulatory functions.

Interestingly, deletion of RORα increased Treg frequencies in vivo. However, treatment with SR3335 trended towards an increase, but did not significantly phenocopy the knock out mice. This may be due, in part, to the pharmacokinetic properties of SR3335 in vivo. The *K*_i_ of SR3335 was reported as 220 nM with an IC_50_ of 480 nM in a Gal4-RORα ligand binding domain cotransfection assay^36^. With the plasma exposure at 230 nM at 6 h and falling quickly thereafter, the activity on target is likely not 100% throughout a 24-h period, even with B.I.D. dosing. Perhaps use of a more potent inverse agonist, with better in vivo exposure, would generate a more significant response and phenocopy the genetic models. Alternatively, a complete deletion of RORα may be necessary to observe the phenotype as changes in chromatin structure are likely to happen in the absence of a gene more so than through pharmacological modification. Regardless, RORα has been demonstrated to be expressed in skin-resident Tregs and its expression is required to restrain allergic skin inflammation^53^. Acquisition of RORα expression appears to occur once the cells are in the skin as RORα expression is low in skin draining lymph nodes^53^. This is intriguing given a previous report described an antagonistic role for Foxp3 in RORα-mediated gene transcription. In this scenario, Foxp3, via its LXXLL domain, directly interacted with RORα to inhibit *Il17a* transcription^10^. While some of the genes differentially expressed in skin-resident RORα-expressing Tregs are similar to those in RORα-deficient T_H_17 cells (*Ccr6*, *Cd44*, *Nr1d1*, etc.), perhaps this molecular antagonism between RORα and Foxp3 may not occur in skin-resident Tregs, or only at select target genes. Based on the increased frequencies of Tregs in RORα-deficient mice with EAE and colitis, this molecular antagonism might occur at sites with different inflammatory stimuli than in the skin. A similar cooperativity has been described for RORγt and STAT3 in the differentiation of T_H_17 vs. Tregs^11^. In the absence of pro-inflammatory cytokines, TGFβ-mediated Foxp3 induction drives Tregs by inhibiting RORγt. However, in the presence of pro-inflammatory cytokines, including IL-6, RORγt is stabilized, promoting T_H_17 differentiation^11^. RORγt-deficient mice have increased Foxp3^+^ Tregs^7^ and are protected from EAE, similar to what we observe in RORα-deficient T cells. It is possible that a similar mechanism occurs between RORα and Foxp3 in the regulation of T_H_17 vs. Treg development, or cooperation occurs between all three transcription factors. However, further in-depth studies are required to understand how RORα may be regulating the T_H_17/Treg balance.

RORα appears to regulate the expression of several genes important for T_H_17 cell migration and function, specifically CCR6^26^. Decreased expression of CCR6 may underlie the decreased number of cells observed in inflamed sites, including the colon and CNS. While CCR6 is classically considered a RORγt target gene^12^, its expression was significantly decreased in vitro and in vivo in RORα-deficient or SR3335-treated T cells. While this may be a consequence of decreased RORγt expression, we do not believe this to be the case because in vitro, protein expression of RORγt present between wild-type and RORα-deficient T_H_17 cells was similar (Fig. 1e). These data would suggest that CCR6 is either a shared target gene with RORγt or, relies on RORα for expression. While *Ccr6* did not appear to be a direct RORα target gene in our analysis, again this could be due to the tissue type analyzed. Interestingly, expression of *Ccr6* is altered in RORα^−/−^ skin Tregs^53^, so we believe the latter is the case. The situation with IL-17A is a little more complicated. It too is decreased in vivo and one could argue that its decreased expression is a function of decreased RORγt expression. However, IL-17A is also decreased in vitro despite equal amounts of RORγt. The answer could lie with the fact that RORγt is a member of the nuclear receptor superfamily and thus a ligand-regulated transcription factor. There have been several reports describing natural, endogenous ligands for RORγt expressed in T_H_17 cells^54–56^. Therefore, expression of a receptor does not always correlate with activity. It is possible that RORα-deficiency leads to dysregulation of pathways that invariably affect the production of endogenous RORγ-ligands. This would manifest as downstream changes in gene expression rather than expression of the receptor itself. Consequently, RORα could affect T_H_17 cell development through mechanisms regulating cellular metabolic processes.

While initially characterized in a diet induced obese (DIO) mouse model of type 2 diabetes^36^, our efforts to examine SR3335’s effects in mouse models of autoimmunity and chronic inflammation have demonstrated that it effectively and largely phenocopies much of the genetic effects observed in the *Rora*^*fl/fl* *×* *CD4 Cre*^ mice. We also found it interesting that SR3335 increased expression of IL-22 in both mouse and human memory T_H_17 cells whereas Nanostring analysis indicated that SR3335 increased the mRNA expression of both *MAF* and *IL22* in human memory T_H_17 and T_H_17.1 cells. This is confounding given that c-Maf has been demonstrated to inhibit IL-22 expression^57^. However, the change in gene expression is minor relative to protein expression via FACS. Given c-Maf’s role in T cell effector function and cytokine expression has been well documented^58–60^, this needs to be interrogated further. This will be particularly important since production of IL-22 by T_H_17 cells has been shown to be upregulated during tissue injury to promote repair^61^. Thus, the observed increase in IL-22 production coupled with decreased IL-17A and TNFα with SR3335 highlight the benefits of targeting RORα in chronic inflammatory diseases like IBD where such actions would prove beneficial to patients. Importantly, the effects on IL-22 appears to be specific to the RORα ligand as other, previously published RORγ inverse agonists did not have similar effects^14^. In contrast to beneficial effects in IBD, SR3335 inhibited TNFα in T_H_17 and T_H_17.1 cells; inhibition of TNF signaling, via use of non-selective TNF inhibitors, exacerbated MS^62,63^ and induced new cases of demyelinating disease and neuropathies in patients treated for other inflammatory diseases^64,65^. These effects were likely due to full abrogation of TNF signaling whereas SR3335 selectively inhibited TNFα in T_H_17 and T_H_17.1 cells, leaving T_H_1 and T_H_2 cells intact. While the effects on TNFα were modest, as were those on IL-22, they need to be further explored to determine if the effects are a cause for concern with potential MS therapies, or the mechanism can be further exploited with more potent RORα inverse agonists for IBD.

While our studies have focused on the positive benefits of inhibiting RORα in T_H_17 cells under chronic inflammatory conditions, tissue-resident “homeostatic” T_H_17 cells play key, non-pathogenic roles in protection of the mucosal barrier^4^. Furthermore, evidence suggests these homeostatic T_H_17 cells may contribute to the development of chronic inflammatory diseases^4^. Therefore, it is possible that T-cell-specific deletion of RORα, and/or inhibition of RORα activity may render the host more susceptible to dissemination of enteric bacteria or infection by pathogenic bacteria, including *Citrobacter rodentium*. However, studies demonstrating transient inhibition or deletion of RORγ activity suggest the host would only be mildly susceptible to *C. rodentium* infection as ILC3s remained intact in mouse hosts^66^. Conversely, a recent report suggests that RORα is required for the maintenance of ILC2s and ILC3s^67^. Given that conditional T-cell deletion of RORα would render ILCs intact and use of a RORα small molecule inhibitor would not completely ablate RORα activity in vivo, it is possible that RORα function would still be sufficient in ILC3s to control *C. rodentium* infection. However, this would have to be tested. Thus, we believe that in the absence of, or inhibition of RORα in T_H_17 cells, the host would be mildly susceptible to bacterial infection at mucosal barriers. Furthermore, a recent report demonstrated that the induction of inflammatory T_H_17 cell responses in the periphery are not influenced by “homeostatic” Th17 cells^68^. This data suggests that any RORα-mediated effects on homeostatic T_H_17 cells would likely have no effect on susceptibility to chronic inflammatory conditions, including EAE. One caveat to these experiments, however, were that they were focused on inflammatory cells generated by *C. rodentium*, not EAE. Therefore, EAE experiments will need to be performed to definitively prove this point.

In summary, we have demonstrated a pathogenic role for RORα in T_H_17 cell development and chronic inflammation. Our work indicates that targeting this receptor for the treatment of T_H_17-mediated autoimmunity may be an alternative strategy that warrants further investigation.

## Methods

### Mice

*C57BL/6*, *SJL/J*, B6.129S7-*Rag1*^*tm1Mom*^/J, stock # 003145 (*Rag1*^*−/*−^), B6(Cg)-Rorc^tm3Litt^/J, stock # 008771 (*Rorc*^*fl/fl*^), and Tg(Cd4-Cre)1Cwi/BfluJ, stock# 017336 (*CD4-Cre)* mice, and B6.SJL-*Ptprc*^*a*^*Pepc*^*b*^*/*BoyJ, stock # 002014 (CD45.1) were purchased from the Jackson Laboratory. The *Rora*^*fl/fl*^ mice were generated on a C57BL/6 background using a previously published targeting strategy: loxp sites were inserted flanking exon 3 of the *Rora* gene, which causes a premature stop codon early in exon 4^69,70^. *Rora*^*fl/fl*^ *×* *CD4-Cre* and *Rorc*^*fl/fl*^ *×* *CD4-Cre* mice were generated by cross breeding *Rora*^*fl/fl*^ or *Rorc*^*fl/fl*^ with *CD4-Cre* mice to produce littermates homozygous for loxp allele and hemizygous for Cre. All groups of experimental mice were matched for age and sex and randomly allocated to treatments within cage groups to eliminate litter effects. All experiments were conducted at controlled temperature (22–23 °C), humidity ~60%, and 12 h:12 h light:dark cycles. Mice had access to regular chow (Harlan 2920X) and water, ad libitum. For all in vitro experiments, mice were killed between 8 and 10 a.m. For all in vivo experiments, mice were killed between 7 and 11 a.m. For EAE experiments, mice were immunized between 11 a.m. and 1 p.m. All mice were maintained under specific pathogen free conditions and the Scripps Florida Institutional Animal Care and Use Committee (IACUC) approved all experiments.

### Human samples

Human blood samples were conducted in accordance with IRB protocols approved by the Institutional Review Board at the Scripps Research Institute (TSRI), OneBlood (Orlando, Florida), and the University of Miami. Blood was obtained following informed written consent from healthy adults or patients with ulcerative colitis. Consenting patients provided clinical history and demographic data at the time of phlebotomy. Institutional review boards at OneBlood and the University of Miami approved all procedures. All forms and documentation that were used obtaining informed consent is stored at OneBlood or the University of Miami. PMBCs were isolated following ficoll density centrifugation and frozen in liquid nitrogen until further analysis. Cryopreserved PBMCs were stored in de-identified and barcoded vials.

### Chemical synthesis and reagents

SR3335^36^ and SR2211^40^ have previously been described. T0901317 was purchased from Cayman Chemical (Cat. #71810) and Rifampicin was purchased from Chem-Impex International, Inc. (Cat. # 00260).

### Mouse in vitro CD4^+^ T cell differentiation

Naïve CD4^+^ T cells from spleen and lymph nodes of male and female 8–10-week-old mice were purified after removing the red blood cells using Lympholyte-M solution (Cedarlane Laboratories). Cells were enriched for naïve CD4^+^ T cells using the mouse naïve CD4^+^ T Cell Isolation Kit (STEMCELL Technologies, Canada) according to the manufacturer’s instruction. If sorting was performed (FACS Aria II; BD Bioscience), the CD4^+^CD25^−^CD62L^hi^CD44^lo^ fraction was collected. The conditions for the different T_H_ cell subsets were: For T_H_0 (neutral conditions): 5 μg/ml anti-IL-4 (clone 11B11, BioLegend) and 5 μg/ml anti-IFNγ (clone XMG1.2, BioLegend); For T_H_1 conditions: 5 μg/ml anti-IL-4, 20 ng/ml IL-12 (R&D Systems), and 10 ng/ml IFNγ (R&D Systems); For T_H_2 conditions: 5 μg/ml anti-IFNγ and 10 ng/ml IL-4 (R&D systems); For T_H_17 conditions: 5 μg/ml anti- IFNγ, 5 μg/ml anti-IL-4 (R&D Systems), and 1.5 ng/ml TGFβ (R&D Systems) and 30 ng/ml IL-6 (R&D Systems); For iT_reg_ conditions: 5 μg/ml anti- IFNγ, 5 μg/ml anti-IL-4, and 5 ng/ml TGFβ (R&D Systems); For Tr1 conditions: 20 ng/mL IL-27 (R & D Systems). Other cytokines used for various T_H_17 conditions: IL-1β (10 ng/ml, R&D Systems) and IL-23 (20 ng/ml, R&D Systems). 1 × 10^6^ cells/ml of naïve CD4^+^ T cells were activated with anti-CD3 (clone 2C11, 0.5 μg/ml) and anti-CD28 (clone 37.51; 1 μg/ml) by precoating plates with 50 μg/ml goat anti-hamster IgG. After 48 h, cells were removed from the TCR signal and recultured at a concentration of 1 × 10^6^cells/ml. Four days after activation, all cells were restimulated with 50 ng/mL phorbol-12-myristate-13-acetate (PMA) (Sigma) and 1 μg/ml ionomycin (Sigma) for 2 h with the addition of GolgiStop (BD Bioscience) for an additional 2 h before intracellular staining. Cells were cultured in IMDM medium (Invitrogen) with 10% FBS, 100 IU/mL penicillin, 100 μg/ml streptomycin, 50 μM β-mercaptoethanol, and 2 mM l-glutamine. All cultures were performed in a volume of 200 μl in 96-well U-bottomed plates. For memory T cell cultures, sorted cells (CCR6^+^ or CCR6^−^) were added to 96-well round bottom tissue culture plates (3 × 10^4^ cells/well) along with anti-CD3/anti-CD28 coated beads (Invitrogen) and recombinant IL-2 (10U/ml;NCI, Biological Resources Branch) for 6 days and treated as described in the text. On day 6, cells were treated as described above for Intracellular cytokine staining.

### Isolation of human PBMCs and T cells

Human blood samples were collected from OneBlood (Orlando, FL) and processed in accordance with protocols approved by Institutional Review Boards at Scripps Florida. Peripheral blood mononuclear cells (PBMCs) were isolated from healthy donor buffy coats by Ficoll-Paque (Sigma) density gradient centrifugation. Naïve CD4^+^ T cells or effector memory CD4^+^ T cells were separated using the EasySep Human Naïve CD4^+^ T cell isolation kit II or EasySep Human Memory CD4^+^ T cell Enrichment Kit, respectively (STEMCELL Technologies, Canada) according to the manufacturer’s instructions. Cell purity of >95% was obtained in the majority of donors for the experiments. For UC/healthy donor samples, frozen PBMCs were thawed and incubated overnight in fresh media. Cell viability was assessed the following day following by isolation of naïve CD4^+^ T cells following methods outlined above.

### Human in vitro CD4^+^ T cell differentiation

Human T_H_17 cell differentiations were performed as follows: naïve CD4^+^ T cells were isolated from buffy coats and cultured in 96-well plates at a density of 2 × 10^5^ per well. Cells were activated with 5 μg/mL plate-bound human anti-CD3 (clone OKT3; Bio X Cell) in complete RPMI^43^. For T_H_17 differentiation, recombinant human IL-23 (50 ng/mL) and IL-1β (50 ng/mL) were added at the start of culture. Sorted effector memory T cell populations were cultured as follows: cells were cultured in DMEM supplemented with 10% FBS, 100 IU/mL penicillin, 100 μg/ml streptomycin, 50 μM β-mercaptoethanol, and 2 mM l-glutamine 10 mM HEPES, 1% non-essential amino acids, and 1%Na pyruvate. Purified T cells were activated with anti-CD3 and anti-CD28-coated beads (Dynabeads; Invitrogen) for 3 days. After 48 h, IL-2 (30 U/mL) was added to the culture. On day 3, anti-CD3/anti-CD28 coated beads were removed using Dynabeads magnets (Invitrogen), and cells were maintained with IL-2 for an additional 3 days^47^. DMSO or SR3335 was added at the start of the cultures.

### Flow cytometry

Surface staining: single cell suspensions prepared from spleen, lymph nodes, CNS, etc. were washed and stained with fluorescence-conjugated antibodies for 20 minutes, washed, then resuspended in FACS buffer (0.5% BSA, 2 mM EDTA in PBS). Intracellular cytokine staining: cells were restimulated with 50 ng/mL PMA and 1 μg/ml ionomycin for 2 h with the addition of GolgiStop (BD Bioscience) for an additional 2 h. Cells were then surface stained using procedures outlined above, fixed and permeabilized using the Foxp3 staining kit (eBioscience). Flow cytometric analysis was performed on a BD LSRII (BD Biosciences) instrument and analyzed using FlowJo software (TreeStar). All antibodies used in experiments are described in Supplementary Table 1.

### FACS sorting of human and mouse effector memory T cells

All FACS sorting was performed using a FACS Fusion (BD). For sorting of human memory T cell subsets from healthy adult PBMCs, enriched memory CD4^+^ T cells (STEMCELL Tech) were stained using the following antibodies: anti-CD4 (BV605); anti-CD25 (PE-CF594); anti-CD45RO (Alexa700); anti-CCR6 (APC); anti-CCR4 (PE-Cy7); anti-CXCR3 (PerCP-Cy5.5); anti-CRTH2, anti-CCR10 (PE; dump); viability (BV510). Stained cells were resuspended in FACS buffer, filtered through a 40-μm filter, and sorted. Sorting of mouse CCR6^+^ cells was performed as follows: T cells were isolated from the spleens and lymph nodes and enriched using CD4^+^ negative isolation kits from STEMCELL Technologies according to the manufacturer’s instructions. Memory (CD25^−^CD62L^lo^CD44^hi^) were further sorted for CCR6 expression by FACS (FACSAria II)^71^. For more information on antibodies, please see Supplementary Table 1.

### Retroviral transduction

To generate the RORα retroviral vector, RORα was inserted into a modified MIGR1 vector (Addgene) with additional multiple cloning sites inserted, using the BamHI site and EcoRI site. MIGR1 empty vector was used as a control. MIGR1 RORγt retroviral construct was a gift from Dan Littman (Addgene Plasmid #24069). Virus production: Plat-E cells (Cell Biolabs, Inc.) were cultured in DMEM containing 10% fetal bovine serum, 2mM L-glutamine, and 1% penicillin/streptomycin at 37 °C under standard culture conditions. Plat-E cells were seeded at 350,000 cells/ml in a 6-well plate the day before transfection. In all, 3 μg total retroviral plasmid DNA (1.5 μg MIGR1 plus 1.5 μg pCL-Eco) was transfected using Fugene6 reagent (Promega) according to manufacturer’s protocol. Viral supernatant was harvested 48 h post-transfection and used immediately for transduction. For retroviral transduction, naïve CD4^+^ T cells were stimulated as indicated with anti-CD3 and anti-CD28 and cultured under T_H_17 conditions. At 24 h post TCR priming, the culture medium was replaced with virus supplemented with 8 μg/ml polybrene. Plates were centrifuged at 1800 rpm for 75 min at 37 °C and then incubated at 37 °C for 3-4 h. After this time, the medium was replaced with the original media removed before addition of virus.

### Induction and clinical evaluation of EAE

Chronic (MOG) EAE was induced in 10-week-old, female *Rora*^*fl/fl*^ or *Rora*^*fl/fl*^ *x Cd4-Cre* mice, or WT mice (drug treatments) using EAE induction kits (EK-2110; Hooke Laboratories, Lawrence, MA, USA) according to manufacturer’s instructions. Briefly, mice were injected subcutaneously over two sites in the flank with 200 μg/mouse of mouse MOG_35–__55_ peptide in an emulsion of Complete Freund’s Adjuvant (CFA), containing killed *M.tuberculosis*, strain H37Ra. Concentration of killed *M.tuberculosis* was adjusted by lot depending on EAE induction and ranged from ~2–5 mg/mL emulsion. Pertussis toxin was dissolved in PBS and injected i.p. at a range of 200–400 ng/mouse 4 h post-immunization (day 0) and 24 h later. The concentration of pertussis toxin used varied by lot and was determined by dose response studies. For relapsing-remitting EAE (R-EAE), 10-week-old, female SJL/J mice were immunized using EAE induction kits (EK-2120; Hooke Laboratories, Lawrence, MA, USA) according to manufacturer’s instructions. Similar induction protocols were used for R-EAE with the exception that 100 μg/mouse of mouse PLP_139–__151_ peptide in an emulsion of Complete Freund’s Adjuvant (CFA), containing 1 mg/mL of killed *M.tuberculosis*, strain H37Ra, was used. Furthermore, 1 injection of 400 ng/mouse of pertussis toxin following immunization (day 0) occurred. As described above, all concentrations were adjusted by lot for consistent EAE induction. Clinical scoring started 7 days post-immunization after which mice were scored daily according to the following criteria: 0, no clinical disease; 1, limp/flaccid tail; 2, limp tail and hind leg weakness; 3, limp tail and complete paralysis of hind limbs; 4, limp tail, complete hind limb and partial front limb paralysis; and 5, quadriplegia or pre-moribund state. Gradations of 0.5 were used when mice exhibited signs that fell between two scores. All scoring was blinded to genotype or treatment and previous scores for each mouse. For drug treatment experiments, SR3335 was dissolved in a 10% DMSO, 10% Tween 80, and 80% H_2_O solution equaling 3 mg/mL and administered intraperitoneally (i.p.) at 30 mg/kg as was vehicle control (10% DMSO, 10% Tween 80, and 80% H2O) twice per day (b.i.d). For chronic MOG-induced EAE, the treatment was started the evening of the immunization and continued for the duration of the experiment. Disease was monitored for 28 days or as indicated in the figure legend. For R-EAE, the treatment was started the after the animals recovered from the first wave of disease and continued for the duration of the experiment. For all b.i.d. dosing, animals were dosed at 7 a.m. and 7 p.m. (lights on/lights off). At the end of the experiment, mice were anaesthetized and transcardially perfused with PBS, and spleen, peripheral lymph nodes together with spinal cords were removed for single cell isolation for FACS analysis.

### T-cell transfer model of colitis

Spleens were collected from 8–10-week-old, female *Rora*^*fl/fl*^ or *Rora*^*fl/fl*^ *×* *Cd4-Cre* mice to sort naïve CD4^+^ T cells. In all, 5 × 10^5^ CD4^+^CD25^−^CD62L^hi^CD44^lo^ cells suspended in PBS were adoptively transferred i.p. (100 μl/mouse) into 8-week-old, female *Rag1*^*−/−*^ recipient mice^72^. For congenic transfers, similar protocols were used with the exception that naïve female CD45.1 CD4^+^ T cells were sorted in lieu of *Rora*^*fl/fl*^ T cells. Post sort, cells were counted, adjusted for cell number, mixed at a 50/50 ratio, and analyzed by flow cytometry to determine equal frequency of the two experimental groups. Once verified, the mixture was injected into female *Rag1*^*−/−*^ recipients. Mice were monitored bi-weekly for body weight change. Beddings were transferred between cages to minimize the impact of microflora on disease development. Mice were killed to assess histological inflammation due to ethical requirements if they reached 80% or less of their original body weight. For the therapeutic evaluation of SR3335 in the T-cell transfer model of colitis, spleens were collected from 8–10-week-old female C57BL/6 mice to sort naïve CD4^+^ T cells as described above. In total, 5 × 10^5^ sorted naïve CD4^+^ T cells were adoptively transferred i.p. (100 μl/mouse) into female *Rag1*^*−/−*^ recipient mice as described above. Three weeks post-transfer, SR3335 was dissolved in a 10% DMSO, 10% Tween 80, and 80% H_2_O solution at 3 mg/mL and administered i.p. at 30 mg/kg as was vehicle control (10% DMSO, 10% Tween 80, and 80% H2O) twice per day (b.i.d). Mice were treated for 2 weeks. After the two-week period, mice were killed to collect spleens, mesenteric lymph nodes, and intestines for FACS analysis.

### Mouse intestine tissue end-point collection

At the termination of the 12-week experiments, the whole colon and ileum (distal 1/3 part of the small intestine) were removed from the mouse. Each segment of the intestine was opened longitudinally, and the fecal contents were gently removed. Colon length and weight were measured and then the colon was dissected in equal halves, designated as ‘proximal’ and ‘distal’ colon and snap frozen in dry ice for protein and RNA analysis.

### Histological assessment of intestinal inflammation

Following overnight fixation in 10% buffered formalin, proximal and distal colon samples were paraffin-embedded, cut in 5 mm sections, and stained with hematoxylin and eosin (H&E). Inflammation was scored in a blinded fashion using a modified scoring system described in Wang et al.^72^. Briefly, each sample was graded on a scale of 0–3 to assess the following four criteria: assessments of markers of severe inflammation (crypt abscesses, ulcers), tissue damage, degree of epithelial hyperplasia and goblet cell depletion, and inflammatory cell infiltration. Scores for each criteria were added to give an overall inflammation score of 0–12.

### Lamina propria mononuclear cell Isolation from mouse intestine

At the termination of the 4–6-week experiments, to collect mononuclear cells from the lamina propria of intestine, mouse ileum or colon were dissected, opened longitudinally, rinsed three times in cold PBS, and then DMEM phenol-free medium with 200 U/mL penicillin and 200 mg/mL streptomycin one time to remove the fecal content. Washed intestines were incubated in DMEM with 1 mM DTT at room temperature (RT) on a rocker for 30 min then in DMEM with 0.5 M EDTA for 30 min at RT to remove the mucus layer. After washing twice in DMEM, intestines were then digested in DMEM with Liberase and DNase I at 37 °C in a shaking water bath for 30 min. The cells were then dislodged from the lamina propria and submucosa by vigorous shaking for 30 s. The supernatant containing leukocytes was pooled together by centrifugation at 500 g for 5 min. Cells were further cleaned up by 30%/70% Percoll (Sigma) gradient separation before further study.

### Analysis of thymocytes

For the measurement of double positive (DP) thymocyte survival kinetics, 7–9-week-old male C57BL/6 mice were treated with compound or vehicle control for 3 days. All compounds were formulated in 15% cremophor at the following concentrations: SR2211 – 2 mg/mL; SR3335 – 3 mg/mL; and administered i.p., b.i.d. Thymii were collected at the termination of the experiment, minced and passed through 70 μm mesh cell strainers. Cell counts were performed prior to cell staining for flow cytometry analysis.

### Quantitative reverse-transcription polymerase chain reaction

Tissue samples (snap frozen and homogenized) were extracted into TRIzol before RNA purification using RNeasy columns (Zymo Research, CA), followed by cDNA synthesis using iScript (BioRad, CA) containing oligo (dT) and random hexamer primers. qPCR SYBR Green (Roche) was used for quantitative polymerase chain reaction using a HT7900 machine (Life Technologies, CA); passive reference dye ROX was used. Primer efficiencies were determined using complementary DNA and primer dilutions for each gene of interest. Primers used to determine gene expressions can be found in Supplementary Table 2. All gene expression data were normalized to the housekeeping gene *β-actin* or *18* *s*.

### Nanostring

Gene expression was quantified using a custom nCounter probe set containing 55 probe pairs with additional probes for positive and negative controls as well as housekeeping genes. (Nanostring Technologies). Briefly, 30,000 T cells were lysed in 5ul of freshly made buffer RLT (Qiagen) containing β-ME. Biotin-conjugated capture probes and fluorescent-barcoded reporter probes were hybridized to cell lysates overnight at 65 °C in a thermocycler. The following day, post-hybridization processing occurred on all lysates which were then run on an nCounter *SPRINT* (Nanostring Technologies) according to the manufacturer’s instructions. Raw data were normalized and analyzed using nSolver software (Nanostring Technologies).

### RNA−sequencing and data analysis

mRNA was extracted from T_H_17 cells on Day 2 (WT vs. RORα KO cells) or Day 3 (MIGR1 transduced cells) of in vitro differentiation. Total RNA was extracted using Qiagen RNeasy kits, quantified using the Qubit 2.0 Fluorometer (Invitrogen, Carlsbad, CA), and run on the Agilent 2100 Bioanalyzer (Agilent Technologies, Santa Clara, CA) for quality assessment. DNase-treated total RNA (300 ng) was depleted of ribosomal RNA (rRNA) using appropriate probes provided by Illumina (TruSeq Total RNA-seq kit) and further assessed on the bioanalyzer to confirm 18S and 28S rRNA peaks are depleted. rRNA-depleted RNA is processed using the TruSeq Stranded Total RNA sample prep kit (Illumina, San Diego, CA). Briefly, RNA samples are chemically fragmented in a buffer containing divalent cations and heating at 94 °C for 8 min. The fragmented RNA is random hexamer primed and reverse transcribed to generate first strand cDNA. The second strand is synthesized after removing the RNA template and incorporating dUTP in place of dTTP. cDNA is then end repaired and adenylated at their 3′ ends. A corresponding ‘T’ nucleotide on the adapters is utilized for ligating the adapter sequences to the cDNA. The adapter-ligated DNA is purified using magnetic Ampure XP beads and PCR amplified using 12–13 cycles to generate the final libraries. The final libraries are size selected and purified using 1.0 × Ampure XP beads to remove any primer dimers. The final library size is typically 200–600 bp with insert sizes ranging from 80 to 450 bp. Final libraries are validated using bioanalyzer DNA chips and qPCR quantified using primers recognizing the Illumina adapters. Libraries are pooled at equimolar ratios, quantified using qPCR (quantification of only the adapter-ligated libraries) and loaded onto the NextSeq 500 flow cell at 1.8 pM final concentration for pair end 75 bp reads. In total, 20–25 million mappable reads per sample were collected. Demultiplexed and quality filtered raw reads (fastq) generated from the NextSeq 500 were trimmed (adapter sequences) using Flexbar 2.4 and aligned to the reference genome using TopHat version 2.0.9^73^. HT seq-count version 0.6.1 was used to generate gene counts and differential gene expression analysis was performed using Deseq2^74^. The normalized gene counts were used to plot the heatmaps using the heatmap function in Prism. To determine enriched functional groups in the RNA-seq data, KEGG pathway analysis was performed using DAVID^75,76^.

### Western blot analysis

T cells were harvested and washed once with phosphate-buffered saline and then incubated for 10 min at 4 °C in 50 μl of TNT lysis buffer (50 mM Tris-Cl, pH 7.5, 150 mM NaCl and 1% Triton X-100) and a complete protease inhibitor mixture (Roche Applied Science). Lysates were then vortexed for 30 s, and then centrifuged at 4 °C for 10 min to clear up. Protein levels in the supernatants were determined using a Coomassie protein assay, ~10 μg of protein from each sample was separated by 10% Tris-glycine gel and then transferred to a polyvinylidene difluoride membrane (Millipore) and immunoblotted with primary antibodies: mouse RORα (CX-16, Santa Cruz, 1:200 dilution), mouse RORγ (Life Technologies, 1:200 dilution), or β-Actin (Cell Signaling Technology, 1:1000 dilution) and horseradish peroxidase-conjugated (HRP) secondary antibodies (Jackson Immunoresearch; Polyclonal Donkey Anti Goat IgG, Cat # 705^−^035-147, Lot # 128117; Polyclonal Donkey Anti-Rat IgG, Cat # 712-135-150, Lot #109032; Polyclonal Goat Anti-Mouse IgG, Cat # 115-035-174, Lot # 143785). All HRP secondary antibodies were used at a 1:10,000 dilution. Detection of the bound antibody by enhanced chemiluminescence was performed according to the manufacturer’s instructions (Santa Cruz).

### Cell culture

HEK293 (American Type Culture Collection) and Plat-E cells (Cell Biolabs, Inc.) were cultured in DMEM (Invitrogen) supplemented with 10% FBS, 2 mM l-glutamine, and 1% penicillin/streptomycin at 37 °C, 5% CO_2_ under standard culture conditions. Mouse lymphocytes were cultured in IMDM medium (Invitrogen) with 10% FBS, 100 IU/mL penicillin, 100 μg/ml streptomycin, 50 μM β-mercaptoethanol, and 2 mM l-glutamine. Human lymphocytes were cultured in RPMI medium (Invitrogen) with 10% FBS, 100 IU/mL penicillin, 100 μg/ml streptomycin, 50 μM β-mercaptoethanol, and 2 mM l-glutamine HEPES, and Na pyruvate.

### Luciferase reporter assays

HEK293 cells were plated 24 h prior to transfection in 96-well plates at a density of 15 × 10^3^ cells/well. Transfections were performed using Lipofectamine 3000 (Invitrogen) according to manufacturer’s protocol. For drug treatments - 16 h post-transfection, cells were treated with vehicle or compound. Twenty-four hours post-treatment luciferase activity was measured using BriteLite (PerkinElmer Life and Analytical Sciences) and read using an Envision multilabel plate reader (PerkinElmer Life and Analytical Sciences). All values were normalized to DMSO to produce fold induction values.

### Statistical analyses

Samples sizes, statistical analysis, and *P* values for every experiment are stated in the figures. Raw data from independent experiments can be found in the Source data. Statistical analyses were performed using GraphPad PRISM version 6 and 8 (GraphPad Software, Inc., La Jolla, CA). Student’s two-tailed *t*-tests were used for comparison between two groups. To compare differences between groups (i.e. EAE or transfer colitis), unpaired two-tailed nonparametric Mann–Whitney *U* tests with a post hoc test were performed as indicated in the Figure legends. *p* < 0.05 was considered significant.

### Reporting summary

Further information on research design is available in the Nature Research Reporting Summary linked to this article.

## Supplementary information

Supplementary Information

Reporting Summary

## Data Availability

Sequence data have been deposited in the Gene Expression Omnibus (GEO) under accession code GSE160327. All other data are in the article and Supplementary Information files or from the corresponding author upon reasonable request. Source data are provided with this paper.

## Source data

Source Data

## References

[1] Milner JD (2008). Impaired T(H)17 cell differentiation in subjects with autosomal dominant hyper-IgE syndrome. Nature.

[2] Puel A (2011). Chronic mucocutaneous candidiasis in humans with inborn errors of interleukin-17 immunity. Science.

[3] de Beaucoudrey L (2008). Mutations in STAT3 and IL12RB1 impair the development of human IL-17-producing T cells. J. Exp. Med..

[4] Stockinger B, Omenetti S (2017). The dichotomous nature of T helper 17 cells. Nat. Rev. Immunol..

[5] Cho JH (2008). The genetics and immunopathogenesis of inflammatory bowel disease. Nat. Rev. Immunol..

[6] Nair RP (2009). Genome-wide scan reveals association of psoriasis with IL-23 and NF-kappaB pathways. Nat. Genet..

[7] Ivanov II (2006). The orphan nuclear receptor RORgammat directs the differentiation program of proinflammatory IL-17+ T helper cells. Cell.

[8] Yang XO (2008). T helper 17 lineage differentiation is programmed by orphan nuclear receptors ROR alpha and ROR gamma. Immunity.

[9] Lee Y (2012). Induction and molecular signature of pathogenic TH17 cells. Nat. Immunol..

[10] Yang XO (2008). Molecular antagonism and plasticity of regulatory and inflammatory T cell programs. Immunity.

[11] Zhou L (2008). TGF-beta-induced Foxp3 inhibits T(H)17 cell differentiation by antagonizing RORgammat function. Nature.

[12] Ciofani M (2012). A validated regulatory network for Th17 cell specification. Cell.

[13] Hamilton BA (1996). Disruption of the nuclear hormone receptor RORalpha in staggerer mice. Nature.

[14] Xiao S (2014). Small-molecule RORgammat antagonists inhibit T helper 17 cell transcriptional network by divergent mechanisms. Immunity.

[15] Zhang Y (2015). GENE REGULATION. Discrete functions of nuclear receptor Rev-erbalpha couple metabolism to the clock. Science.

[16] Araujo, L., Khim, P., Mkhikian, H., Mortales, C. L. & Demetriou, M. Glycolysis and glutaminolysis cooperatively control T cell function by limiting metabolite supply to N-glycosylation. *Elife*10.7554/eLife.21330 (2017). 10.7554/eLife.21330 PMC5257256 28059703

[17] Berod L (2014). De novo fatty acid synthesis controls the fate between regulatory T and T helper 17 cells. Nat. Med..

[18] Gerriets VA (2015). Metabolic programming and PDHK1 control CD4+ T cell subsets and inflammation. J. Clin. Invest..

[19] Shi LZ (2011). HIF1alpha-dependent glycolytic pathway orchestrates a metabolic checkpoint for the differentiation of TH17 and Treg cells. J. Exp. Med..

[20] Xu T (2017). Metabolic control of TH17 and induced Treg cell balance by an epigenetic mechanism. Nature.

[21] Shin B (2020). Mitochondrial oxidative phosphorylation regulates the fate decision between pathogenic Th17 and regulatory T cells. Cell Rep..

[22] Wu L (2020). Niche-selective inhibition of pathogenic Th17 cells by targeting metabolic redundancy. Cell.

[23] Eftekharian MM (2016). RAR-related orphan receptor A (RORA): A new susceptibility gene for multiple sclerosis. J. Neurol. Sci..

[24] McGeachy MJ, Cua DJ (2007). The link between IL-23 and Th17 cell-mediated immune pathologies. Semin. Immunol..

[25] McGeachy MJ (2007). TGF-beta and IL-6 drive the production of IL-17 and IL-10 by T cells and restrain T(H)-17 cell-mediated pathology. Nat. Immunol..

[26] Reboldi A (2009). C-C chemokine receptor 6-regulated entry of TH-17 cells into the CNS through the choroid plexus is required for the initiation of EAE. Nat. Immunol..

[27] Liston A (2009). Inhibition of CCR6 function reduces the severity of experimental autoimmune encephalomyelitis via effects on the priming phase of the immune response. J. Immunol..

[28] Morrison PJ (2013). Th17-cell plasticity in Helicobacter hepaticus-induced intestinal inflammation. Mucosal Immunol..

[29] Lee YK (2009). Late developmental plasticity in the T helper 17 lineage. Immunity.

[30] Hirota K (2011). Fate mapping of IL-17-producing T cells in inflammatory responses. Nat. Immunol..

[31] Bending D (2009). Highly purified Th17 cells from BDC2.5NOD mice convert into Th1-like cells in NOD/SCID recipient mice. J. Clin. Invest..

[32] Harbour SN, Maynard CL, Zindl CL, Schoeb TR, Weaver CT (2015). Th17 cells give rise to Th1 cells that are required for the pathogenesis of colitis. Proc. Natl Acad. Sci. USA.

[33] Habtezion A, Nguyen LP, Hadeiba H, Butcher EC (2016). Leukocyte trafficking to the small intestine and colon. Gastroenterology.

[34] Cosmi L (2008). Human interleukin 17-producing cells originate from a CD161+CD4+ T cell precursor. J. Exp. Med..

[35] Annunziato F (2007). Phenotypic and functional features of human Th17 cells. J. Exp. Med..

[36] Kumar N (2010). Identification of SR3335 (ML-176): a synthetic RORalpha selective inverse agonist. ACS Chem. Biol..

[37] Repa JJ (2000). Regulation of absorption and ABC1-mediated efflux of cholesterol by RXR heterodimers. Science.

[38] di Masi A, De Marinis E, Ascenzi P, Marino M (2009). Nuclear receptors CAR and PXR: Molecular, functional, and biomedical aspects. Mol. Aspects Med..

[39] Amir M (2018). REV-ERBalpha regulates TH17 cell development and autoimmunity. Cell Rep..

[40] Kumar N (2012). Identification of SR2211: a potent synthetic RORgamma-selective modulator. ACS Chem. Biol..

[41] El-Behi M (2011). The encephalitogenicity of T(H)17 cells is dependent on IL-1- and IL-23-induced production of the cytokine GM-CSF. Nat. Immunol..

[42] Codarri L (2011). RORgammat drives production of the cytokine GM-CSF in helper T cells, which is essential for the effector phase of autoimmune neuroinflammation. Nat. Immunol..

[43] Revu S (2018). IL-23 and IL-1beta drive human Th17 cell differentiation and metabolic reprogramming in absence of CD28 costimulation. Cell Rep..

[44] Acosta-Rodriguez EV (2007). Surface phenotype and antigenic specificity of human interleukin 17-producing T helper memory cells. Nat. Immunol..

[45] Sallusto F, Zielinski CE, Lanzavecchia A (2012). Human Th17 subsets. Eur. J. Immunol..

[46] Nistala K (2010). Th17 plasticity in human autoimmune arthritis is driven by the inflammatory environment. Proc. Natl Acad. Sci. USA.

[47] Ramesh R (2014). Pro-inflammatory human Th17 cells selectively express P-glycoprotein and are refractory to glucocorticoids. J. Exp. Med..

[48] Girardi CS (2019). Nuclear RXRalpha and RXRbeta receptors exert distinct and opposite effects on RA^−^mediated neuroblastoma differentiation. Biochim. Biophys. Acta Mol. Cell Res..

[49] Ramon-Vazquez A (2019). Common and differential transcriptional actions of nuclear receptors LXRalpha and LXRbeta in macrophages. Mol. Cell. Biol..

[50] Shimada K (2018). T-cell-intrinsic receptor interacting protein 2 regulates pathogenic T helper 17 cell differentiation. Immunity.

[51] Chung Y (2009). Critical regulation of early Th17 cell differentiation by interleukin-1 signaling. Immunity.

[52] Ikeda S (2014). Excess IL-1 signaling enhances the development of Th17 cells by downregulating TGF-beta-induced Foxp3 expression. J. Immunol..

[53] Malhotra, N. et al. RORalpha-expressing T regulatory cells restrain allergic skin inflammation. *Sci. Immunol.*10.1126/sciimmunol.aao6923 (2018). 10.1126/sciimmunol.aao6923 PMC5912895 29500225

[54] Hu X (2015). Sterol metabolism controls T(H)17 differentiation by generating endogenous RORgamma agonists. Nat. Chem. Biol..

[55] Santori FR (2015). Identification of natural RORgamma ligands that regulate the development of lymphoid cells. Cell Metab..

[56] Soroosh P (2014). Oxysterols are agonist ligands of RORgammat and drive Th17 cell differentiation. Proc. Natl Acad. Sci. USA.

[57] Rutz S (2011). Transcription factor c-Maf mediates the TGF-beta-dependent suppression of IL-22 production in T(H)17 cells. Nat. Immunol..

[58] Neumann C (2019). c-Maf-dependent Treg cell control of intestinal TH17 cells and IgA establishes host-microbiota homeostasis. Nat. Immunol..

[59] Gabrysova L (2018). c-Maf controls immune responses by regulating disease-specific gene networks and repressing IL-2 in CD4(+) T cells. Nat. Immunol..

[60] Xu M (2018). c-MAF-dependent regulatory T cells mediate immunological tolerance to a gut pathobiont. Nature.

[61] Brockmann, L., Giannou, A. D., Gagliani, N. & Huber, S. Regulation of TH17 cells and associated cytokines in wound healing, tissue regeneration, and carcinogenesis. *Int. J. Mol. Sci.*10.3390/ijms18051033 (2017). 10.3390/ijms18051033 PMC5454945 28492497

[62] van Oosten BW (1996). Increased MRI activity and immune activation in two multiple sclerosis patients treated with the monoclonal anti-tumor necrosis factor antibody cA2. Neurology.

[63] TNF neutralization in MS: results of a randomized, placebo-controlled multicenter study. (1999). The Lenercept Multiple Sclerosis Study Group and The University of British Columbia MS/MRI Analysis Group. Neurology.

[64] Sicotte NL, Voskuhl RR (2001). Onset of multiple sclerosis associated with anti-TNF therapy. Neurology.

[65] Richez C, Blanco P, Lagueny A, Schaeverbeke T, Dehais J (2005). Neuropathy resembling CIDP in patients receiving tumor necrosis factor-alpha blockers. Neurology.

[66] Withers DR (2016). Transient inhibition of ROR-gammat therapeutically limits intestinal inflammation by reducing TH17 cells and preserving group 3 innate lymphoid cells. Nat. Med..

[67] Lo BC (2019). The transcription factor RORalpha preserves ILC3 lineage identity and function during chronic intestinal infection. J. Immunol..

[68] Omenetti S (2019). The intestine harbors functionally distinct homeostatic tissue-resident and inflammatory Th17 cells. Immunity.

[69] Dussault I, Fawcett D, Matthyssen A, Bader JA, Giguere V (1998). Orphan nuclear receptor ROR alpha-deficient mice display the cerebellar defects of staggerer. Mech. Dev..

[70] Billon C, Sitaula S, Burris TP (2017). Metabolic characterization of a novel RORalpha knockout mouse model without ataxia. Front. Endocrinol..

[71] Carlson TJ (2014). Halofuginone-induced amino acid starvation regulates Stat3-dependent Th17 effector function and reduces established autoimmune inflammation. J. Immunol..

[72] Wang R (2015). Neutralizing IL-23 is superior to blocking IL-17 in suppressing intestinal inflammation in a spontaneous murine colitis model. Inflamm. Bowel Dis..

[73] Trapnell C, Pachter L, Salzberg SL (2009). TopHat: discovering splice junctions with RNA^−^Seq. Bioinformatics.

[74] Anders S, Huber W (2010). Differential expression analysis for sequence count data. Genome Biol..

[75] Huang da W, Sherman BT, Lempicki RA (2009). Systematic and integrative analysis of large gene lists using DAVID bioinformatics resources. Nat. Protoc..

[76] Huang da W, Sherman BT, Lempicki RA (2009). Bioinformatics enrichment tools: paths toward the comprehensive functional analysis of large gene lists. Nucleic Acids Res..

