# Supplementary material for: Genetic and pharmacological inhibition of the nuclear receptor RORα regulates TH17 driven inflammatory disorders
Source: Nat Commun. 2021 Jan 4;12:76. doi: 10.1038/s41467-020-20385-9 (PMC7782731; doi:10.1038/s41467-020-20385-9)
Supplement: Supplementary file 1 — Supplementary Information [file 41467_2020_20385_MOESM1_ESM.pdf]

SUPPLEMENTARY INFORMATION

Genetic and pharmacological inhibition of the nuclear receptor RORα regulates T_H17 driven inflammatory disorders

Ran Wang, Sean Campbell, Mohammed Amir, Sarah A. Mosure, Molly A. Bassette, Amber Eliason, Mark S. Sundrud, Theodore M. Kamenecka, and Laura A. Solt

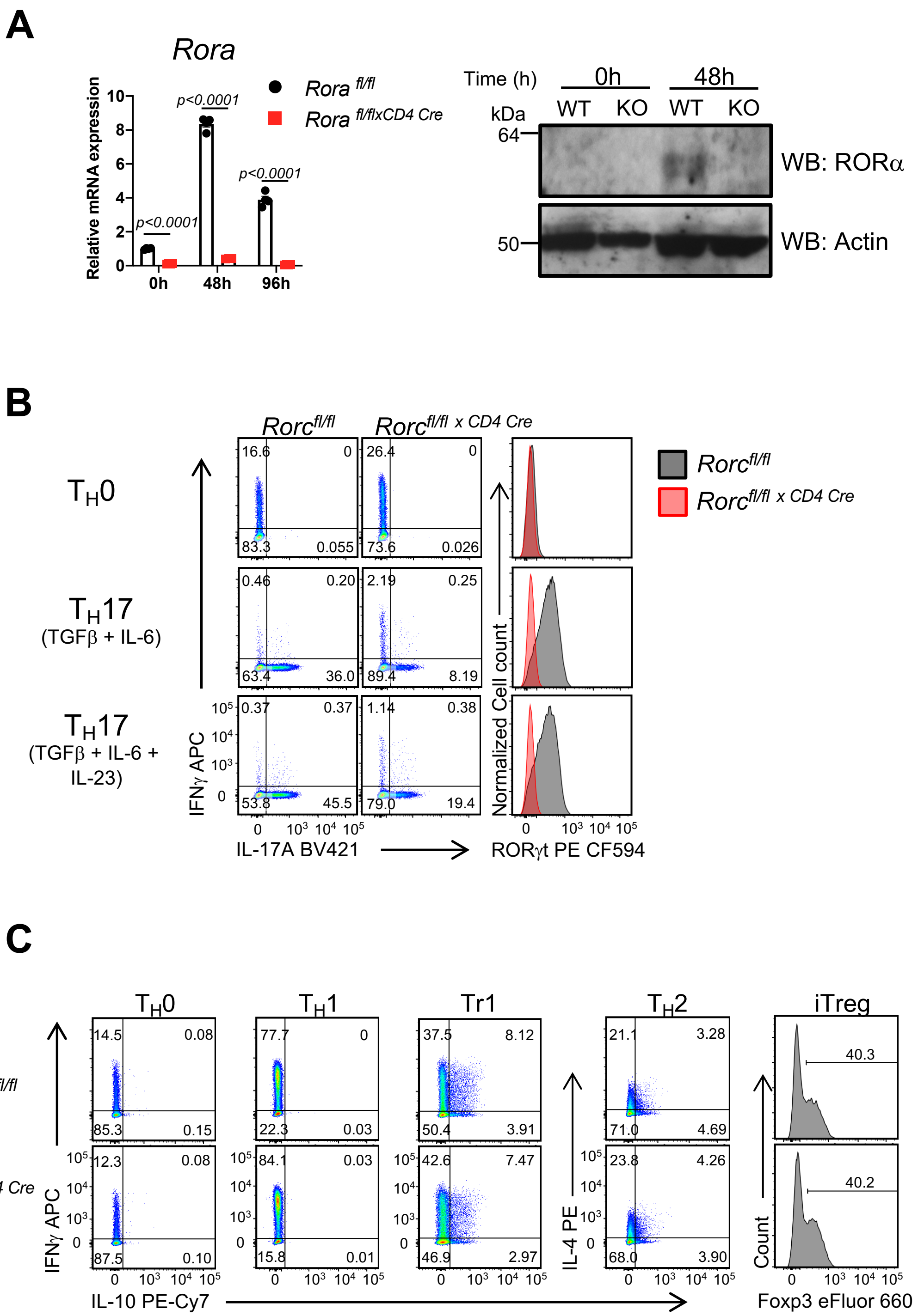

Supplementary Figure 1. **Loss of RORα does not effect other $CD4^+$ T helper cell populations *in vitro*.** **(A)** qRT-PCR (*left panel*) and western blot (*right panel*) of naive $CD4^+$ T cells differentiated into T_H17 cells from $Rora^{fl/fl}$ (WT) or $Rora^{fl/fl\ x\ CD4\ Cre}$ (KO) mice demonstrating RORα is deleted from T_H17 cells. Uncropped blots in Source Data. **(B)** FACS analysis of naive $CD4^+$ T cells differentiated into T_H0 or T_H17 cells (non-pathogenic vs pathogenic) from $Rorc^{fl/fl}$ or $Rorc^{fl/fl\ x\ CD4\ Cre}$ mice. **(C)** FACS analysis of naive $CD4^+$ T cells differentiated into T_H0, T_H1, Tr1, T_H2, or iTregs cells from $Rora^{fl/fl}$ or $Rora^{fl/fl\ x\ CD4\ Cre}$ mice. Data are presented as mean values ±s.e.m. Data is from one of three separate experiments demonstrating similar results. Student's two-tailed *t*-tests were performed for statistical analysis.

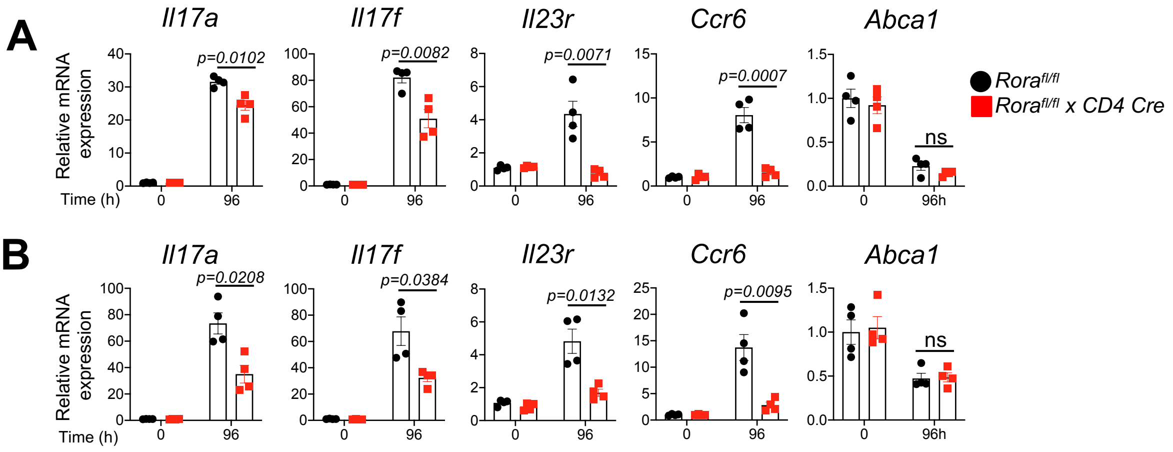

<u>Supplementary Figure 2</u>. Loss of RORα affects the T_H17 developmental program under various cytokine conditions. qRT-PCR of T_H17-mediated cytokines in T_H17 cell cultures **(A)** (TGFβ + IL-6 + IL-23) or **(B)** (IL-1β + IL-6, + IL-23) from WT and KO mice. β-actin was used as the internal control. Data are presented as mean values ±s.e.m. (n=4). Student's two-tailed *t*-tests were performed for statistical analysis. ns, not significant (*p>0.05*).

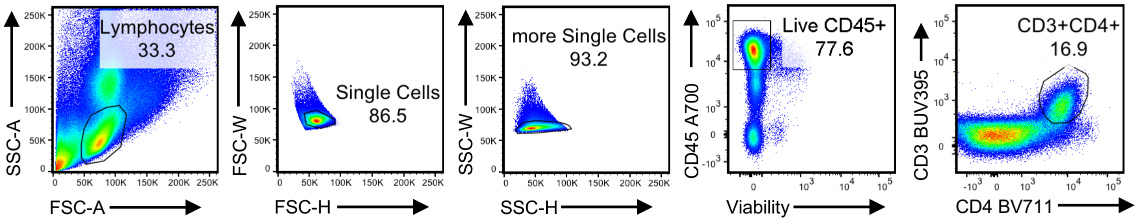

<u>Supplementary Figure 3</u>. Gating strategy for *in vivo* FACS analysis experiments.

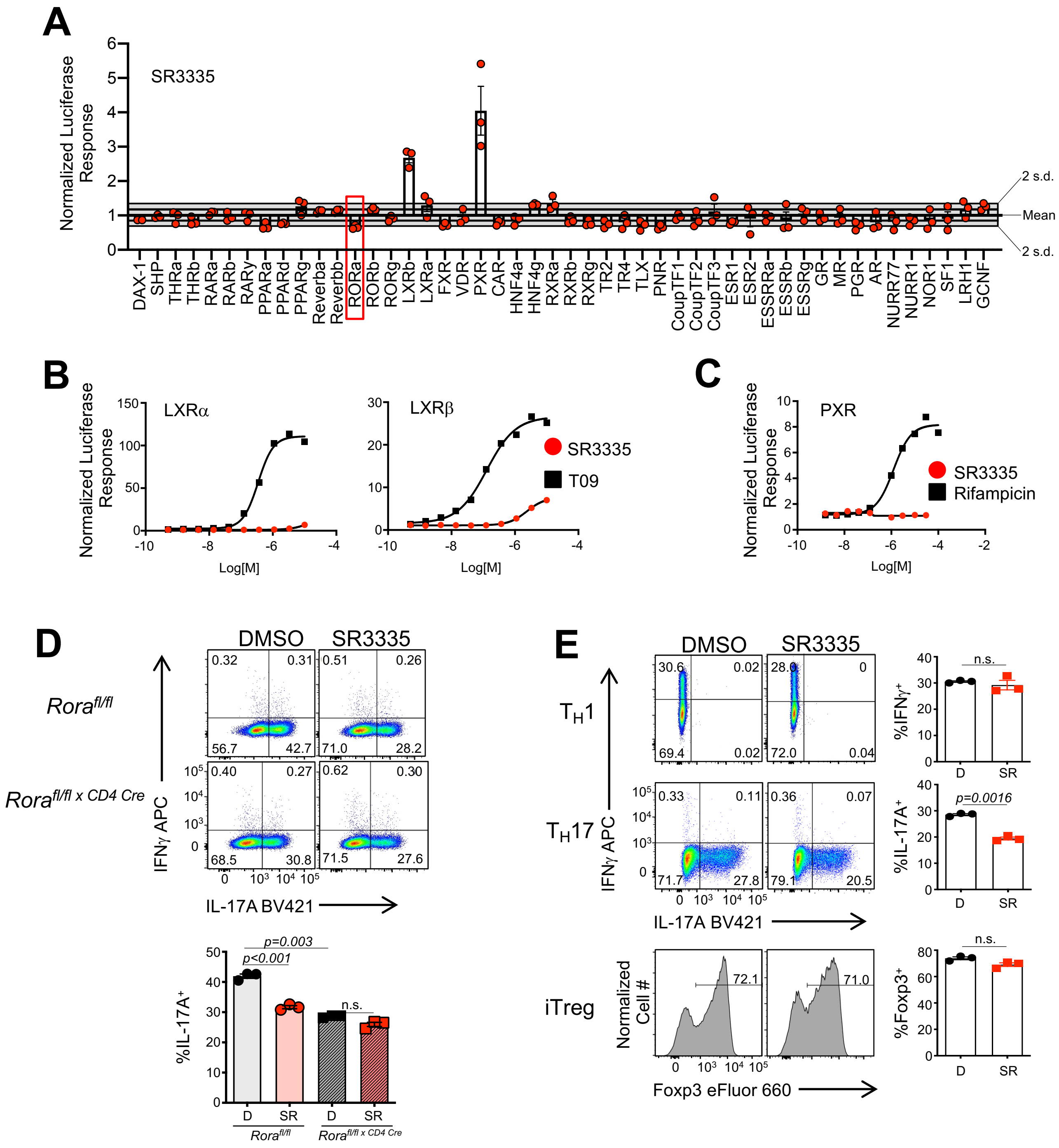

Supplementary Figure 4. **SR3335 is selective for RORα. (A)** Nuclear receptor specificity assay illustrating the activity of SR3335 (10μM) against all 48 human nuclear receptors represented in the panel. HEK293 cells were co-transfected with GAL4 DNA binding domain-NR LBD fusion constructs as previously described. *(n=3)*. **(B & C)** Dose response analysis of SR3335 vs the lead compound, T09, at GAL4 LXRα and LXRβ **(B)** or the PXR agonist, Rifampicin, at GAL4 PXR **(C)**. *(n=8)*. **(D)** Specificity of SR3335 (10μM) was assessed in T_H17 cells from *Rora^fl/fl^* and *Rora^fl/fl x CD4 Cre^* mice. FACS analysis of IL-17A and IFNγ expression was performed 96h post T-cell activation. Data is from one of three separate experiments demonstrating similar results. Graph (below) summarizes data from one of the experiments in which several mice from each group were used. (**E**) FACS analysis and graphs depicting percent cultures (top), IL-17A and IFNγ expression from T_H1 (top), T_H17 (middle), and $Foxp3^+$ cells in iTreg cell culture conditions (bottom) in the presence of vehicle (DMSO) or SR3335 (5μM). Data are presented as mean values ±s.e.m. Data are representative of three separate independent experiments generating similar results. Student's two-tailed *t*-tests were performed for statistical analysis. ns, not significant (*p>0.05*).

A

Time (h) post injection	[Plasma] (μM)
0.25	24.67 +/- 0.50
0.5	30.12 +/- 0.36
1	28.59 +/- 2.26
2	17.76 +/- 1.93
4	1.29 +/- 0.18
6	0.23 +/- 0.03
8	0.08 +/-0.01
24	0.002 +/- 0.0007

SR3335, 30 mg/kg i.p., 3 mg/mL solution
In 10/10/80 DMSO/Tween80/water

B

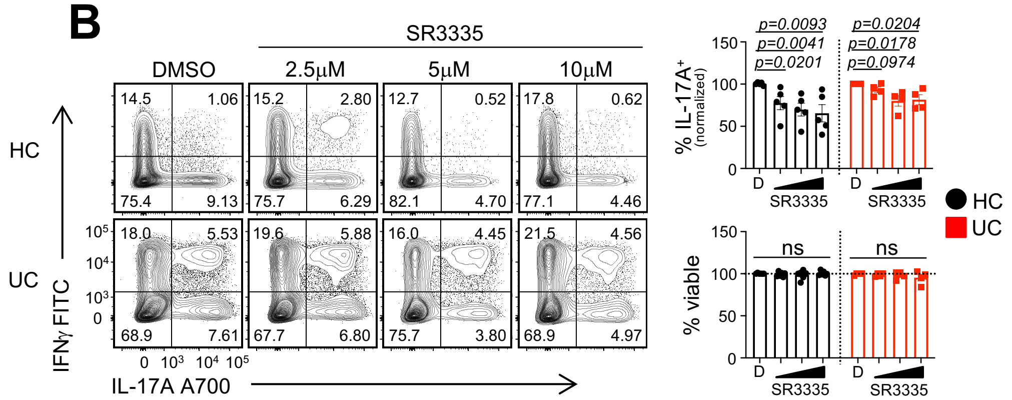

C

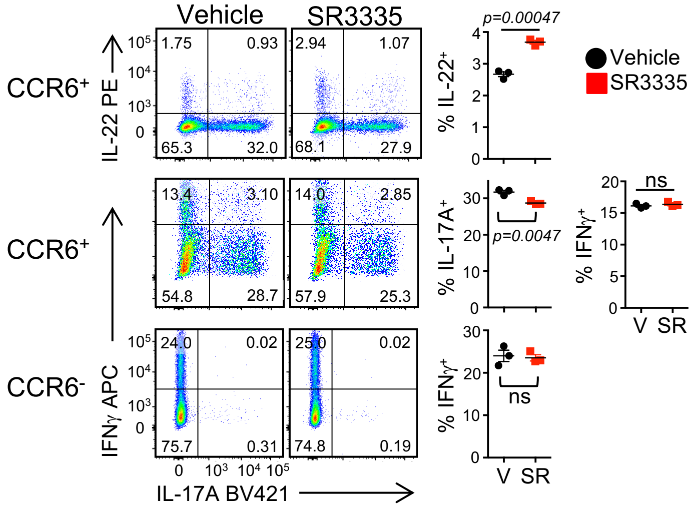

Supplementary Figure 5. **SR3335 modulates cytokine expression in human T_H17 cells from UC patients and from mouse memory T_H17 cells. (A)** Pharmacokinetic analysis of SR3335 in mice. Data represents mean±s.e.m (n=3). (**B**) Naive human CD4+ T cells were isolated from PBMCs from healthy donors (HC) or Ulcerative colitis (UC) patients and differentiated under T_H17 polarizing conditions (anti-CD3+IL-23+IL-1β) and treated with vehicle (DMSO) or 3 different concentrations of SR3335. IL-17A and IFNγ expression were analyzed from live, CD45RO+ cells by flow cytometry on Day 6. Graphs indicate percent IL-17A+ cells (top, normalized to DMSO) and frequency of live cells (bottom, normalized to DMSO) in cultures with compound treatment. (HC, n=5 biologically independent samples; UC, n=4 biologically independent samples) (**C**) Intracellular FACS analysis of sorted CCR6+ and CCR6- cells that were stimulated with anti-CD3/anti-CD28 beads and cultured for 6 days (+IL-2) in the presence of vehicle or SR3335 (5μM). On day 6 cells were restimulated with PMA and Ionomycin in order to determine IL-17A, IL-22, and IFNγ expression. Graphs on the right summarize the data from one experiment. Data are presented as mean values ±s.e.m. Data are representative of three separate experiments generating similar results (n=3 technical replicates/group). Student's two-tailed *t*-tests were performed for statistical analysis. ns, not significant.

Antibodies used for mouse experiments						
**Antibody**	**Fluorophore**	**Species**	**Isotype**	**Clone**	**Company**	**Cat #**
$\alpha 4 \beta 7$	PE	Rat	IgG2a, kappa	DATK32	Biolegend	120605
CD3e	BUV395	Hamster	IgG	145-2C11	BD	563565
CD4	BV711	Rat	IgG2a, kappa	RM4-5		100549
CD8a	FITC	Rat	IgG2a, kappa	53-6.7	eBioscience	11-0081-85
CD19	FITC	Rat	IgG2a, kappa	eBio 1 D3	eBioscience	11-0193-86
CD25	BV605	Rat	IgG1, lambda	PC61	Biolegend	102035
CD44	PerCP-Cy5.5	Rat	IgG2b, kappa	IM7	Biolegend	103031
CD45	A700	Rat	IgG2b, kappa	30-F11	Biolegend	103128
CD45.1	FITC	Mouse	IgG2a, kappa	A20	Biolegend	110713
CD62L	BV421	Rat	IgG2a, kappa	MEL14	BD	562910
CD196(CCR6)	eFluor 660	Rat	IgG2a, kappa	sirx6	eBioscience	50-7196-80
CXCR3	PECy7	Armenian Hamster	IgG	CXCR3-173	Biolegend	126515
B220	FITC	Rat	IgG2a, kappa	RA3-6B2	eBioscience	11-0452-82
FoxP3	eFluor 660	Rat	IgG2a, kappa	FJK-16s	eBioscience	50-5773-82
GM-CSF	PE	Rat	IgG2a, kappa	MP1-22E9	Biolegend	505405
IL-4	PE	Rat	IgG1, kappa	11B11	Biolegend	504103
IL-10	PE-Cy7	Rat	IgG2b, kappa	JES5-16E3	Biolegend	505025
IL-17A	BV421	Rat	IgG1, κ	TC11-18H10	BD	563354
IL-17F	PE	Rat	IgG2a, kappa	eBio18F10	eBioscience	12-7471-82
IL-22	PE	Goat	Polyclonal IgG	Poly5164	Biolegend	516404
IFNg	PE-Cy7	Rat	IgG1, kappa	XMG1.2	eBioscience	25-7311-82
IFNg	APC	Rat	IgG1, kappa	XMG1.2	eBioscience	17-7311-82
RORgt	PE CF-594	Mouse	IgG2a, κ	Q31-378	BD	562684
Viability	eFluor 780				eBioscience	65-0865-14
Viability	eFluor 506				eBioscience	65-0866-14

Antibodies used for human experiments						
**Antibody**	**Fluorophore**	**Species**	**Isotype**	**Clone**	**Company**	**Cat #**
CD45RO	A700	mouse	mouse IgG2a, k	UCHL1	Biolegend	304218
CD4	BV605	mouse	mouse IgG2b, k	OKT4	Biolegend	317437
CD25	PE CF-594	mouse	mouse IgG1, k	M-A251	BD	562525
CCR6	APC	mouse	mouse IgG2b, k	G034E3	Biolegend	353415
CCR4	PE-Cy7	mouse	mouse IgG1, k	L291H4	Biolegend	359409
CXCR3	PerCP-Cy5.5	mouse	mouse IgG1, k	G025H7	Biolegend	353713
CRTH2	PE	Rat	Rat IgG2a, k	BM16	Biolegend	350105
CCR10	PE	Armenian Hamster	IgG	6588-5	Biolegend	341503
IL-2	Pe-Cy7	Rat	Rat IgG2a, k	MQ1-17H12	Biolegend	500325
IL-4	PerCP-Cy5.5	Rat	Rat IgG1, k	MP4-25D2	Biolegend	500821
IL-13	PE	Rat	Rat IgG, k	JES10-5A2	Biolegend	501903
IL-17A	A700	mouse	mouse IgG1, k	BL168	Biolegend	512317
IL-22	APC	mouse	mouse IgG2a, k	2G12A41	Biolegend	366705
IFNg	FITC	mouse	mouse IgG1, k	4S.B3	Biolegend	502505
TNFa	BV421	mouse	mouse IgG1, k	MAb11	Biolegend	502931
Viability	eFluor 506				eBioscience	65-0866-14

Supplementary Table 1. Antibodies used for Flow cytometry experiments.

qRT-PCR primers		
**Gene name**	**Forward primer (5' - 3')**	**Reverse Primer (5' - 3')**
Abca1	GGACATGCACAAGGTCCTGA	CAGAAAATCCTGGAGCTTCAAA
b-actin	CCACAGCTGAGAGGGAAATC	AAGGAAGGCTGGAAAAGAGC
18s	GTAACCCGTTGAACCCCATT	CCATCCAATCGGTAGTAGCG
Ccr6	TCCAGGCAACCAAATCTTTC	AGGGCTTGAGATGATGATGG
Csf2	TGGAAGCATGTAGAGGCCATCA	GCGCCTTG AGTTTGGTGAAAT
Cxcl10	TCCTTGTCCTCCCTAGCTCA	ATAACCCCTTGGGAAGATGG
Ifng	TGGCTGTTTCTGGCTGTTACT	GCTCTGCAGGATTTTCATGTC
Il1b	GTTTTCCTCCTTGCCTCTGA	GCTGCCTAATGTCCCCTT
Il6	CATGTTCTCTGGGAAATCGTG	TCCAGTTTGGTAGCATCCATC
Il17a	CTCCAGAAGGCCCTCAGACTAC	AGCTTTCCCTCCGCATTGACACAG
Il17f	GAGGATAACACTGTGAGAGTTGAC	GAGTTCATGGTGCTGTCTTCC
Il21	CAGATCGCCTCCTGATTAGACT	CTCACAGTGCCCCTTTACATC
Il22	TCATCGGGGAGAAACTGTTC	CATGTAGGGCTGGAACCTGT
Il23p19	CTTCTCCGTTCCAAGATCCTTCG	GGCACTAAGGGCTCAGTCAGA
Il23r	TGTGGATCCTGTCCTTACAGAG	CCTAGAGGACAGTCTCTTGTCTCA
Rora	AACCCGAACCCATATGTGAC	ATGTTCTGGGCAAGGTGTTC
Rorc	CCGCTGAGAGGGCTTCAC	TGCAGGAGTAGGCCACATTACA
Tnfa	TCAGCCGATTTGCTATCTCAT	TGGAAGACTCCTCCCAGGTAT

Supplementary Table 2. Primers used for qRT-PCR.
