# Supplementary material for: Genetic and pharmacological inhibition of the nuclear receptor RORα regulates TH17 driven inflammatory disorders
Source: Nat Commun. 2021 Jan 4;12:76. doi: 10.1038/s41467-020-20385-9 (PMC7782731; doi:10.1038/s41467-020-20385-9)
Supplement: Supplementary file 2 — Reporting Summary [file 41467_2020_20385_MOESM2_ESM.pdf]

nature research

Corresponding author(s): Laura A. Solt

Last updated by author(s): 2020 - 10-29

Reporting Summary

Nature Research wishes to improve the reproducibility of the work that we publish. This form provides structure for consistency and transparency in reporting. For further information on Nature Research policies, see Authors & Referees and the Editorial Policy Checklist .

Statistics

For all statistical analyses, confirm that the following items are present in the figure legend, table legend, main text, or Methods section.

n/a	Confirmed	
☐	☒	The exact sample size (*n*) for each experimental group/condition, given as a discrete number and unit of measurement
☐	☒	A statement on whether measurements were taken from distinct samples or whether the same sample was measured repeatedly
☐	☒	The statistical test(s) used AND whether they are one- or two-sided<br>*Only common tests should be described solely by name; describe more complex techniques in the Methods section.*
☒	☐	A description of all covariates tested
☐	☒	A description of any assumptions or corrections, such as tests of normality and adjustment for multiple comparisons
☐	☒	A full description of the statistical parameters including central tendency (e.g. means) or other basic estimates (e.g. regression coefficient) AND variation (e.g. standard deviation) or associated estimates of uncertainty (e.g. confidence intervals)
☐	☒	For null hypothesis testing, the test statistic (e.g. *F*, *t*, *r*) with confidence intervals, effect sizes, degrees of freedom and *P* value noted<br>*Give P values as exact values whenever suitable.*
☒	☐	For Bayesian analysis, information on the choice of priors and Markov chain Monte Carlo settings
☒	☐	For hierarchical and complex designs, identification of the appropriate level for tests and full reporting of outcomes
☒	☐	Estimates of effect sizes (e.g. Cohen's *d*, Pearson's *r*), indicating how they were calculated

Our web collection on statistics for biologists contains articles on many of the points above.

Software and code

Policy information about availability of computer code

Data collection	The following software was used to collect data: BD FACSDIVA software (v9) for FACS, RNA-sequencing: TopHap v 2.0.9., HT-seq count v 0.61, Deseq2 (v1.18.1).
Data analysis	Methods describe the data analysis for the RNA-sequencing including DAVID Bioinformatics Resources v6.8, GraphPAD Prism6 and 8 for statistics, nSolver software v4.0 for Nanostring, and for FlowJo v10 for FACS analysis.

For manuscripts utilizing custom algorithms or software that are central to the research but not yet described in published literature, software must be made available to editors/reviewers. We strongly encourage code deposition in a community repository (e.g. GitHub). See the Nature Research guidelines for submitting code & software for further information.

Data

Policy information about availability of data

All manuscripts must include a data availability statement. This statement should provide the following information, where applicable:

- Accession codes, unique identifiers, or web links for publicly available datasets
- A list of figures that have associated raw data
- A description of any restrictions on data availability

Source data are provided with this paper. All next-generation sequencing data generated for this paper have been deposited in the Gene Expression Omnibus (GEO) under accession number GSE160327. All other data are available from the corresponding author upon reasonable request.

Field-specific reporting

Please select the one below that is the best fit for your research. If you are not sure, read the appropriate sections before making your selection.

☒ Life sciences ☐ Behavioural & social sciences ☐ Ecological, evolutionary & environmental sciences

For a reference copy of the document with all sections, see nature.com/documents/nr-reporting-summary-flat.pdf

Life sciences study design

All studies must disclose on these points even when the disclosure is negative.

Sample size	Sample size was selected based on 1) previously published experimental data (Amir et al., Cell Rep. 2018; Wang et al., Inflamm Bowel Dis. 2015) and 2) maximum probability of uncovering statistical significance between 2 groups.
Data exclusions	No data were excluded
Replication	Each experiment used new groups/cohorts of animals, with consistent results. All replicates are stated in figure legends as either biological or technical replicates. Each in vitro experiment was repeated at least 3 times, each in vivo experiment was repeated at least twice if the two experiments repeated with consistent results.
Randomization	For in vivo studies, age and sex matched animals were assigned randomly to each experimental and control group where applicable. For in vitro studies, randomization was not relevant since pools of cells, using equal numbers between groups within an experiment, was used for studies.
Blinding	The assessment of EAE scores was performed in a blinded fashion. Experimental analysis of samples from all in vivo experiments (i.e. FACS, tissues for qRT-PCR, histology) was performed in blinded manner (i.e. samples were labeled 1, 2, 3, etc., analyzed, and groups uncovered once analysis was completed). All other experimental techniques were not blinded. Blinding in other experiments was not necessary as readouts occurred either via machine (ie Flow cytometer) or using a method that was not arbitrary (ie weight as a measurement).

Reporting for specific materials, systems and methods

We require information from authors about some types of materials, experimental systems and methods used in many studies. Here, indicate whether each material, system or method listed is relevant to your study. If you are not sure if a list item applies to your research, read the appropriate section before selecting a response.

Materials & experimental systems

n/a	Involved in the study	
☐	☒	Antibodies
☐	☒	Eukaryotic cell lines
☒	☐	Palaeontology
☐	☒	Animals and other organisms
☐	☒	Human research participants
☒	☐	Clinical data

Methods

n/a	Involved in the study	
☒	☐	ChIP-seq
☐	☒	Flow cytometry
☒	☐	MRI-based neuroimaging

Antibodies

Antibodies used	See Supplementary Table 1 for FACS antibodies. Western blot antibodies: anti RORa - Santa Cruz BioTech, CX-16; anti RORgt (AFKJS-9) -Life Technologies (Cat # 14-6988-82); b-Actin (8H10D10) - Cell Signaling Technology - Cat #3700S). Horseradish peroxidase-conjugated secondary antibodies (Jackson Immunoresearch; Polyclonal Donkey Anti Goat IgG, Cat# 705-035-147, Lot # 128117; Polyclonal Donkey Anti-Rat IgG, Cat# 712-135-150, Lot #109032; Polyclonal Goat Anti-Mouse IgG, Cat# 115-035-174, Lot # 143785). In vitro T helper cell differentiation antibodies: anti IFNg - BioLegend, clone 11B11, Cat# 504135; anti-IL-4, BioLegend, clone XMG1.2, Cat# 505847, anti-CD3 (clone 145-2C11, BioLegend, Cat# 100340); anti CD28 (clone 37.51, BioLegend, Cat # 112116), human anti CD-3 (clone OKT3, Bio X Cell);
Validation	All antibodies were validated for the species (either human or mouse) and application by the manufacturer, which is described on the manufacturer's website.

Eukaryotic cell lines

Policy information about cell lines

Cell line source(s)	HEK293 (ATCC); PlatE cells (Cell Biolabs, Inc)

Authentication	Authenticated using STR.
Mycoplasma contamination	All cells were tested and found to be mycoplasma free.
Commonly misidentified lines (See ICLAC register)	No commonly misidentified cell lines were used in this study

Animals and other organisms

Policy information about studies involving animals; ARRIVE guidelines recommended for reporting animal research

Laboratory animals	C57BL/6, SJL/J, Rag1-/- (B6.129S7-Rag1 tm1Mom/J, stock# 003145, Jackson labs), Rorc floxed mice (B6(Cg)-Rorc tm3Litt/J, stock# 008771, Jackson labs), Cd4-Cre (Tg(Cd4-Cre)1Cwi/BfluJ), stock# 017336, Jackson labs), Rora floxed mice, B6.SJL-PtprcaPepcb/BoyJ, stock # 002014 (CD45.1) . For in vitro experiments, both male and female mice were used - between 8-10 weeks old. For in vivo experiments, either female, age, and littermate matched mice between 8-11 weeks of age or male mice, age and littermate matched were used.
Wild animals	No wild animals were used in this study
Field-collected samples	No field collected samples were used in this study.
Ethics oversight	All animal work was approved by the Scripps Florida Institutional Animal Care and Use Committee (IACUC).

Note that full information on the approval of the study protocol must also be provided in the manuscript.

Human research participants

Policy information about studies involving human research participants

Population characteristics	Consenting patients provided clinical history and demographic data at the time of phlebotomy.<br>UC patients (n=4): Mean age, years (SD) 37 (16); Sex, male/female (%) 50/50; Ethnicity (% Hispanic/Non-Hispanic): 50/50; Treatment categories: anti-TNF/other (%) 50/50<br>Healthy donors (n=5): Mean age, years (SD) 38 (13); Sex, male/female (%) 40/60; Ethnicity (% Hispanic/Non-Hispanic): 40/60
Recruitment	Recruitment of Ulcerative Colitis patients was performed following informed written consent at the University of Miami in Miami, Florida. No selection was performed, all patients were recruited. Healthy donors were recruited internally at the University of Miami or from OneBlood (Orlando, Florida). Selection was based on availability and consent.
Ethics oversight	All experiment using human blood or PBMCs was conducted in accordance with IRB protocols by the Scripps Research Institute or the University of Miami.

Note that full information on the approval of the study protocol must also be provided in the manuscript.

Flow Cytometry

Plots

Confirm that:

- [x] The axis labels state the marker and fluorochrome used (e.g. CD4-FITC).
- [x] The axis scales are clearly visible. Include numbers along axes only for bottom left plot of group (a 'group' is an analysis of identical markers).
- [x] All plots are contour plots with outliers or pseudocolor plots.
- [x] A numerical value for number of cells or percentage (with statistics) is provided.

Methodology

Sample preparation	Spleens, lymph nodes, CNS, intestinal tissues were gently macerated under a nylon mesh strainer using the flat end of a 3-mL syringe. Red blood cells were removed using Lympholyte-M or Percoll (see Methods for complete details). Cells were washed with FACS buffer, filtered, pelleted, and stained for FACS.
Instrument	BD LSR II for sample analysis, BD FACS ARIA for cell sorting.
Software	FlowJo 10 (Tree Star) for FACS analysis. FACSDIVA for sample collection.
Cell population abundance	The purities of sorted cells were 99%+.
Gating strategy	For in vitro cell differentiations, based on the pattern of FSC-A/SSC-A, cells in the lymphocyte gate were used followed by isolation of singlets (based on FSC-H vs FSC-W; SSC-H vs SSC-W) and viable cells. Positive populations were determined by the specific antibodies, which were distinct from the negative populations. For the in vivo experiments, a similar gating strategy was

used, with the exception that live, CD45+ cells were gated on prior to identifying CD3+CD4+ populations. An example of an in vivo gating strategy is shown in supplemental information.

☒ Tick this box to confirm that a figure exemplifying the gating strategy is provided in the Supplementary Information.
